# Allosteric Communication in the Tubulin Dimer Revealed through Molecular Dynamics and Network Analysis

**DOI:** 10.1021/acsomega.6c08528

**Published:** 2026-10-11

**Authors:** Polina Teterina, Christine Peter

**Affiliations:** Department of Chemistry, University of Konstanz, Universitätsstraße 10, Konstanz 78457, Germany

## Abstract

Microtubules are dynamic polymers essential for intracellular organization, with basic constituents being αβ-tubulin dimers. Microtubule growth and shrinkage is tightly regulated by GTP hydrolysis in β-tubulin, yet how the information about the bound nucleotide propagates across the tubulin dimer, beyond its direct binding site, remains unclear. Here we combine extensive all-atom molecular dynamics simulations of αβ-tubulin dimers with GTP or GDP in the β-subunit to investigate longitudinal allosteric signaling. We featurize conformations using residue-wise minimum distances between the two subunits, apply EncoderMap dimensionality reduction and HDBSCAN clustering to identify conformational substates, and perform dynamic network analysis on these clusters. We find that the tubulin dimer relaxes from the straight starting structures into a broad ensemble of bent conformations, and that the bound nucleotide does not by itself separate this ensemble. The simulations retain a comparatively long-lived memory of their starting structure that makes the subtler nucleotide signal harder to detect. Network analysis identifies residues that are most important for information flow, as well as preferred communication routes across the longitudinal interface. Substate-specific analysis indicates that the transition from the GTP- to GDP-bound state is associated with changes in the preferred allosteric routes, enhancing our understanding of longitudinal communication in tubulin. Beyond tubulin, our approach illustrates how combining dimensionality reduction with substate-specific network analysis can uncover hidden allosteric signatures in protein assemblies.

## Graphical abstract

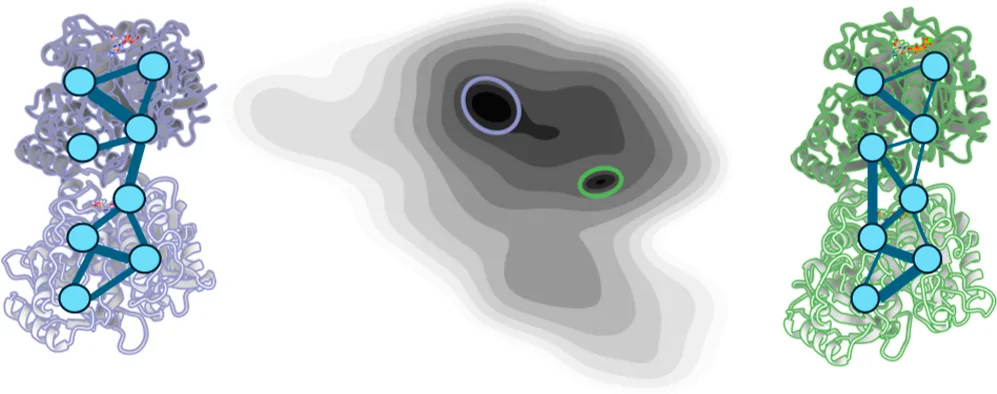

## 1 Introduction

Tubulin αβ heterodimers form hollow tubes of larger polymer structures, microtubules, which among other vital cellular functions separate the chromosomes apart during cell division^1^ and dictate the intracellular organization of the cells.^2^ Within the microtubule, dimers polymerize head-to-tail into protofilaments through longitudinal contacts, while neighboring protofilaments associate side-by-side through lateral contacts to close the tube wall. Experimental observations of microtubule ends show their dramatic structural rearrangements in space and time when they alter stochastically between periods of growth and shrinkage. This behavior is rooted in the intrinsic dynamics of αβ-tubulin. In the dimer both subunits have nucleotide-binding sites. The α-subunit permanently binds guanosine triphosphate (GTP) which is located in the intradimer longitudinal interface. In contrast, GTP in the β-subunit is readily hydrolyzed to GDP after a new heterodimer binds on top of it. The incoming dimer provides residues that complete the GTPase active site of the β-tubulin, making it catalytically competent.^3^ The nucleotide bound to the β-subunit is referred to as the nucleotide state of the tubulin dimer. Following the hydrolysis of GTP to GDP in the β-subunit, microtubules become prone to depolymerization. This process, called dynamic instability,^4^ is a crucial part of the dynamic nature of microtubules and enables them to generate mechanical forces during assembly and disassembly and to physically probe the cytoplasm to locate cellular targets.^5^ The fact that nucleotide hydrolysis governs this process suggests its far-reaching conformational influence that extends beyond the β-tubulin binding pocket - even across the intradimer protein–protein interface. Such coupled conformational changes propagating through protein structures are at the heart of allostery.^6,7^

Proteins are intrinsically flexible molecular machines whose function often depends on transitions between multiple conformational substates.^8^ Molecular dynamics (MD) simulations provide a natural way to capture this structural diversity, generating high-dimensional data sets that describe the equilibrium fluctuations of the system.^9^ Decoding this conformational phase space typically involves featurizing each structure with suitable descriptors (for example, a set of distances or angles) and projecting them onto low-dimensional maps,^10^ where clustering can identify metastable and functionally relevant substates. In parallel to such geometric analyses, graph theory has emerged as a powerful framework for studying long-range allosteric communication.^11–13^ By representing residues as nodes and their interactions as weighted edges, network analysis provides a view of “information flow” within a protein, revealing communication pathways and central residues important for allosteric signal transduction.^14^ However, constructing these residue-interaction networks requires careful methodological choices: how nodes and edges are defined, how contact maps or correlation metrics (linear or generalized) are computed, and, critically, how simulation frames are combined into a single graph. Simple averaging across heterogeneous MD trajectories can blur state-specific allosteric couplings, underscoring the need for protocols that explicitly respect the underlying conformational diversity.^15^

Previous computational atomistic and multiscale studies of tubulin dimers indicate that the nucleotide state modulates dimer mechanics and interfacial flexibility.^16–18^ Network studies on tubulin have so far mostly focused on lateral communication. Basu et al.^19^ used dynamical network and community analysis to compare tubulin with and without a bound taxane, which is a class of microtubule-stabilizing anticancer drugs, such as Taxol, that bind a pocket in the β-subunit and affect lateral contacts within the microtubule.^20^ They found that taxanes binding increases the connectivity between the communities on the two lateral contact surfaces of the β-subunit. Extending to the microtubule lattice, Cannariato et al.^21^ applied MD-based network analysis and found that the seam, the single lattice discontinuity where lateral contacts are of the opposite type, weakens lateral transfer between protofilaments. Taken together, these studies suggest that tubulin communication is malleable in the presence of ligands and in different lattice geometries. What remains less explored, however, is the other direction, whether the nucleotide state alone can reshape intradimer longitudinal communication.

In this study, we aim to describe information transfer across the longitudinal intradimer interface. To do so, we first characterize the conformational landscape of the αβ-tubulin dimer and its heterogeneity. We then identify distinct, conformationally homogeneous substates and use them as a basis for network analysis. By doing so, rather than building networks from entire trajectories, we avoid averaging distinct substates together. Regarding the tubulin system specifically, we explore longitudinal information transfer, in contrast to previous studies that focused on lateral communication.

## 2 Results and Discussion

We performed extensive all-atom MD simulations of the αβ-tubulin dimer. The starting conformations were taken from two experimental cryo-EM microtubule-lattice structures that were solved with different nucleotides bound: PDB 3J6E, obtained with the GTP analogue GMPCPP, and PDB 3J6F, obtained with GDP.^22^ To disentangle the effect of the bound nucleotide from that of the starting conformation, we simulated dimers with each nucleotide from both starting structures. We placed either GDP or GTP in the β-subunit pocket (systems we refer to as GDP- and GTP-tubulin, Figure 1a). We call a simulation matched when the nucleotide state is the same as in the experimental structure it was built from (GDP in 3J6F, GTP in 3J6E) and swapped otherwise (GDP in 3J6E, GTP in 3J6F). In addition, we ran apo simulations, with no nucleotide in the β-subunit, from both structures. Matched trajectories are labeled G­[D/T]­P[1–6] and swapped ones G­[D/T]­P[1–6]_sw (Table 1). To get a better visual understanding of the dimer with its structural moieties, we refer to Figure S1.

**Figure 1:**
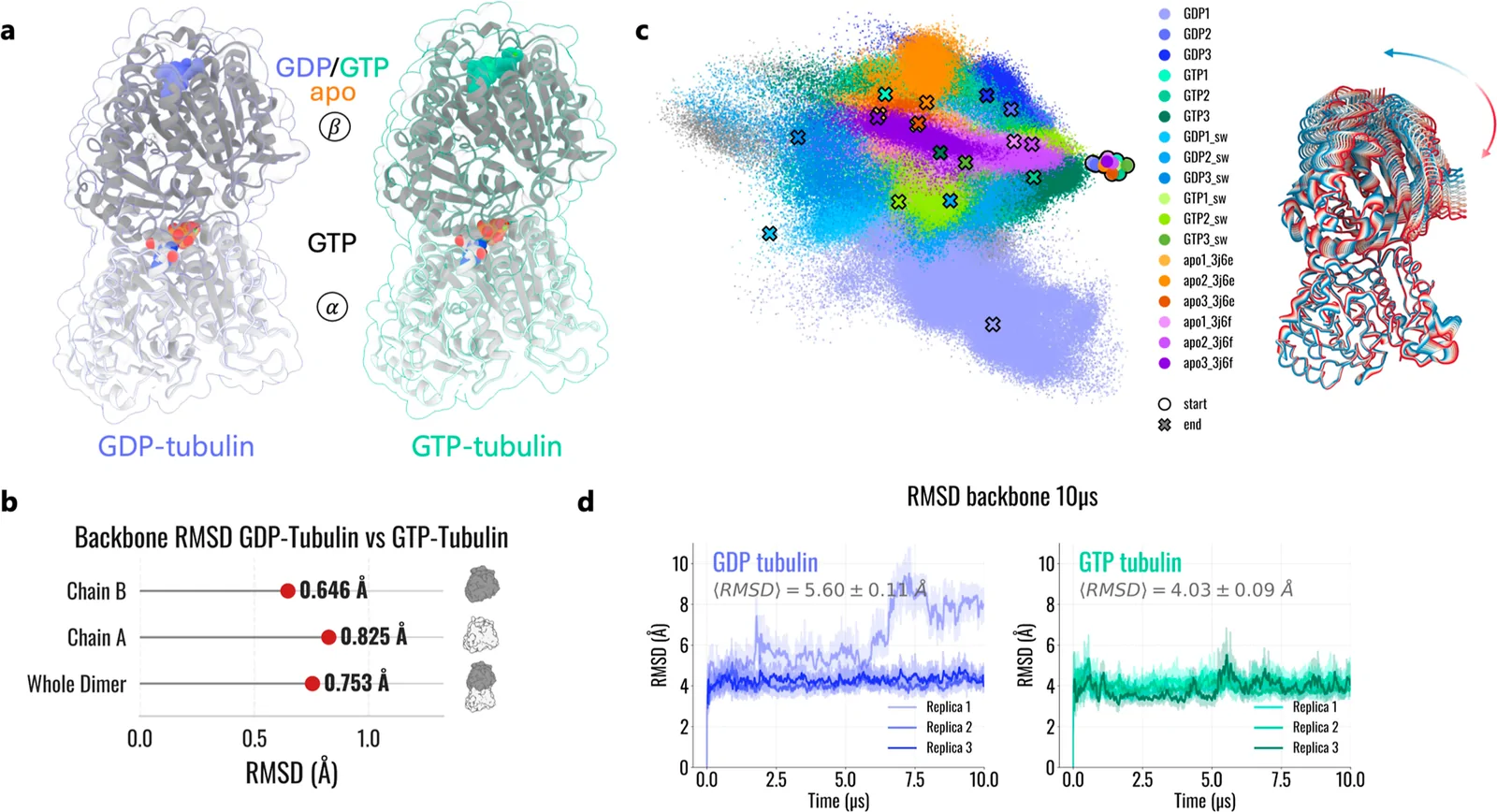
(a) Starting structures for GDP- and GTP-tubulin dimers (the surface outline depicts the protein conformation extracted from the corresponding microtubule lattice PDB IDs 3J6E (green) and 3J6F (blue)).^22^ The nucleotide in the β-subunit (GDP/GTP) and the nonexchangeable GTP in the α-subunit are shown as spheres. (b) Backbone RMSD between the initial protein conformations 3J6F vs 3J6E for the individual monomers and for the whole dimer. (c) Conformational landscape obtained from projection of residue-wise minimum distances of all simulations (coloring by replica, for clarity not all 30 replicas are displayed in color here, for the rest see Figure S2). Starting structures are indicated by black-edged circles and final structures by black-edged crosses. The structure on the right illustrates the dominant bending motion from a principal component analysis of the Cartesian Cα coordinates^19^ (red and blue: extreme conformations, arrows: direction of motion). (d) Backbone RMSD of the three 10 μs GDP (left, blue) and GTP (right, green) matched replicas, computed relative to the starting structure. For each replica the solid line is a running average over 30 ns with the full data shown transparently in the same color. Additionally, the plot shows the mean ⟨RMSD⟩ ± standard error for *n* = 3 replicas for the last 1 μs of simulations.

**Table 1:** Summary of MDs Simulation Systems

label	ligand in the β-subunit	protein conformation	time (μs)
GDP[1–3]	GDP	3J6F	3 × 10
GTP[1–3]	GTP	3J6E	3 × 10
GDP[4–6]	GDP	3J6F	3 × 3
GTP[4–6]	GTP	3J6E	3 × 3
GDP[1–6]_sw	GDP	3J6E	6 × 2
GTP[1–6]_sw	GTP	3J6F	6 × 2
apo_[1–3]_3j6e	no ligand	3J6E	3 × 1
apo_[1–3]_3j6f	no ligand	3J6F	3 × 1

Before applying the network analysis to the simulation data we set out to characterize the extent and overlap of the conformational sampling of the MD trajectories. Therefore, to assess global conformational sampling, we first chose a set of internal high-dimensional descriptors for the two domain protein system. The descriptors are sets of residue-wise minimum distances (RMDs) between α-subunit and β-subunit, which have been found to be well suited for the description of topological relationships between protein domains or subunits^23^ (for more details see the methods section). To visualize the behavior of the system, we projected the high-dimensional data of all simulations into a two-dimensional plane resulting in the global conformational landscape (Figure 1c). More details about this landscape will be discussed in Section 2.1 below. Here the landscape serves as an illustration whether during the time evolution of the separate simulations several replicas were visiting similar states and whether the GTP and GDP system behaved differently (for the part of the landscape covered by each individual simulation see Figure S2). Overall, all trajectories appear to move through a central region of the conformational landscape before fanning out to a varying extent. In particular, GDP1 behaves unusually, occupying a large region in the lower part of the landscape. We observe that during the GDP1 replica, the ligand dissociates from the β-tubulin pocket. The special status of the GDP1 simulation is further supported by an assessment of the structural stability via the RMSD metric (Figure 1d). For the simulation where the GDP dissociates, the RMSD values increase to more than 8 Å. The underlying reason for this special behavior will be discussed in Section 2.3. The increase of the RMSD values of the other replicas likewise indicates a relaxation of the dimer from the initial straight conformation, leveling off at a very similar value of approximately 4 Å. Given that the two initial structures differ by less than 1 Å, this substantially larger range suggests that any initial differences are rapidly superseded by structural relaxation of the dimer during the early stages of the simulations. In order to improve the quality of the sampling and add further simulation data with fully bound nucleotide in the β-tubulin subunit, we performed another set of three replicas each for GDP- and GTP-tubulin of 3 μs length—in addition to the first set of 10 μs long simulations. Additionally, as mentioned above, control simulations with the nucleotide swapped relative to the starting structure were performed, as well as apo simulations without a nucleotide bound to the β-tubulin subunit. Backbone RMSD data for all 30 simulations is provided in Figure S3. Overall, the simulations cover (6 × 10 μs) + (6 × 3 μs) + (12 × 2 μs) + (6 × 1 μs) = 108 μs.

### 2.1 Conformational Landscape Reflects Bending of the Tubulin Dimer

Next, to get a deeper insight into the visited phase space of mutual subunit–subunit configurations and to prepare representative structures for the network analysis the two-dimensional conformational landscape is explored in more detail. As mentioned, dimensionality reduction typically requires an input feature set of internal coordinates.^10^ For a structure with two subunits such as the tubulin dimer, RMDs make up a suitable set of internal coordinates that scales linearly with the number of protein residues. The RMDs are defined as follows: for every residue in one subunit the minimum distance to any atom in the other subunit is calculated and vice versa. RMDs have been found to be input features that very well capture patterns in protein–protein interactions since they are highly sensitive to the contact interface but also include longer-ranged distance information that encodes the relative orientation of the two subunits.^23,24^ Thus, RMDs between the two subunits are selected here to describe changes in the shape of the tubulin dimer and the relative orientation of the subunits in the different dimer conformations. Therefore, for every frame of the trajectory we have an 864-dimensional set of internal coordinates, that includes minimum distances for 438 residues in the α-subunit (to the β-subunit) and minimum distances for 426 residues in the β-subunit (to the α-subunit).

To this data set, we applied a nonlinear dimensionality reduction algorithm, EncoderMap, to produce a low-dimensional conformational landscape. EncoderMap consists of an autoencoder architecture with an additional Sketchmap-based^25^ loss function which can emphasize particular intervals of pairwise (dis)­similarities between the data points, for example, to reduce the spread of similar conformations due to thermal fluctuations.

Figure 1c displays the EncoderMap projection of the whole set of 30 simulations. Coloring the map based on the specific condition (Figure 2a–c) shows that simulations started from different starting structures for the GDP/GTP/apo states occupy overlapping but shifted regions. Specifically, the GTP matched and swapped sets overlap substantially (Figure 2a, with an overlap coefficient of 0.42), whereas the GDP sets (Figure 2b, with an overlap coefficient of 0.28) overlap less along the horizontal axis. The apo simulations overlap to an intermediate degree (Figure 2c, with an overlap coefficient of 0.36) and only cover a narrower area of the central region of the map. This is probably due to the fact that they are shorter (1 μs each) than the other simulations. Importantly, the starting structure changes not only the extent of the sampled landscape but also the density distribution. The high-density core regions (the innermost contours) of the matched and swapped sets are displaced relative to each other. The fact that simulations initiated from different starting structures continue to occupy partially distinct regions of the conformational landscape indicates incomplete convergence of the simulations with respect to the subunit–subunit orientation on the available time scales. The position of the starting structures on the map (Figure 1c) (depicted as black-edged circles), which are bundled together at the far right, indicates how different the conformational ensemble of the dimer is in solution compared to the microtubule lattice where the dimer has to have straight conformation. After the start of the simulation the dimer relaxes to more curved conformations.

**Figure 2:**
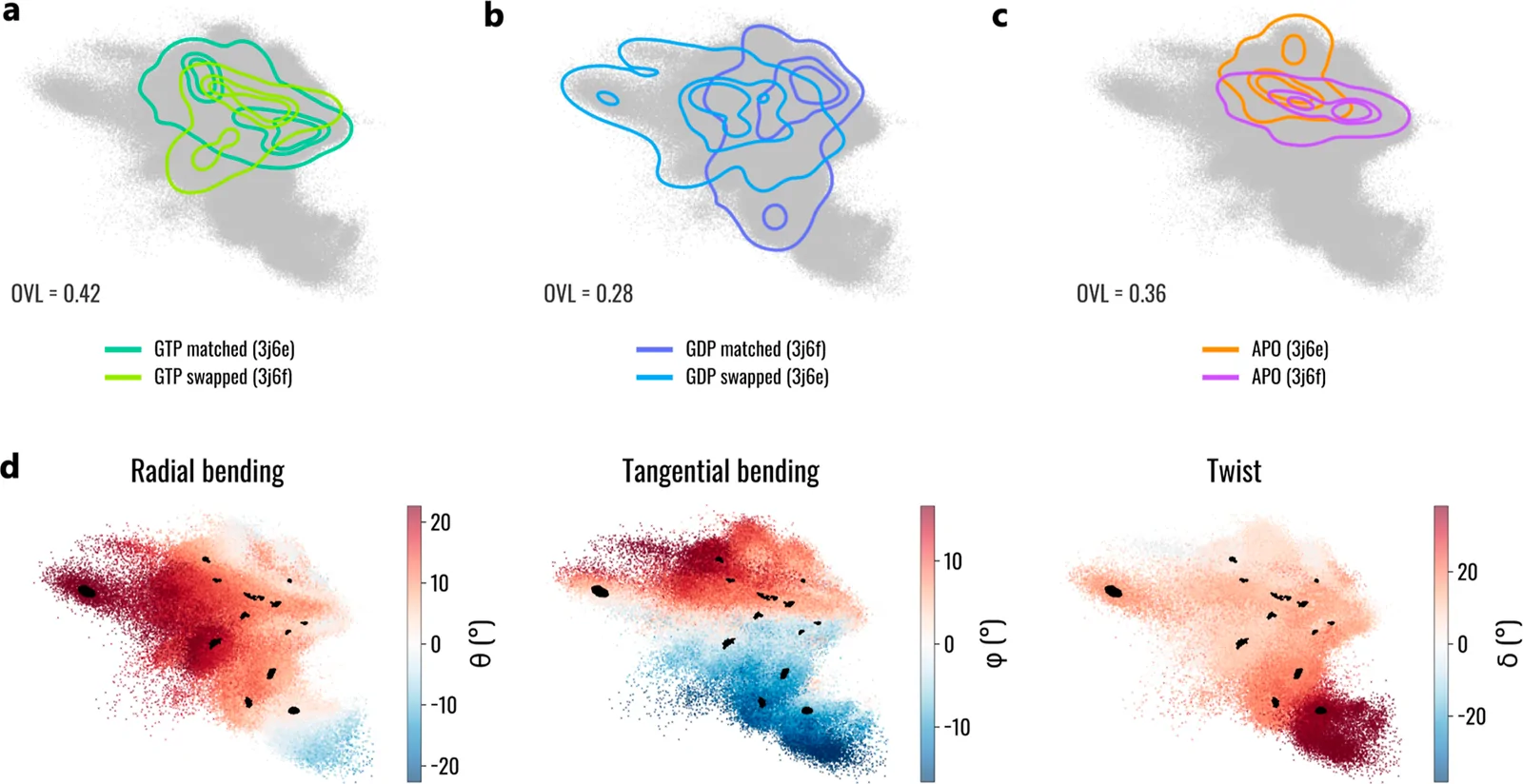
(a–c) Conformational landscape with density contour lines enclosing 98%, 60% and 30% of each subset’s density, for simulations started from the matched versus swapped starting structure: (a) GTP matched (3J6E) vs GTP swapped (3J6F); (b) GDP matched (3J6F) vs GDP swapped (3J6E); (c) apo started from 3J6E vs 3J6F. In (b), frames of the GDP1 replica after GDP dissociated from the nucleotide-binding pocket are excluded from the density contours. OVL stands for the overlap coefficient and represents the fraction of density shared by the two subsets, where 1 would indicate identical distributions. Gray points show the projection of the entire data set. (d) The same landscape colored by the value of the three intradimer bending angles—radial (θ), tangential (ϕ) and twist (δ). The diverging color scale is centered at 0° (the straight initial geometry), so red and blue mark opposite bending directions. Black points indicate the position of the HDBSCAN cluster exemplars, discussed in Section 2.3.

In order to better understand the meaning of the different regions of the map—which should by construction be sensitive to the dimer interface—the simulation data and the landscape were inspected with respect to the intradimer orientation. For that, we inspected the bending of the α-subunit relative to the β-subunit with the help of three rotation angles. These angles were defined following a previously described procedure^26^ where an internal coordinate system within the tubulin subunits is defined that is associated with the microtubule lattice wall. The three angles describe how the internal coordinate systems of the two subunits are aligned/rotated with respect to each other. In this system, the *z*-axis extends along the microtubule axis (rotation around this axis corresponds to a twisting motion), the *x*-axis is tangential to the microtubule surface (rotation around it corresponds to radial bending, where 0° represents the straight lattice geometry and positive values indicate curvature away from the microtubule axis), and the *y*-axis is radial to the microtubule (corresponding to the tangential component of bending). For a graphical illustration see Figure S4.

Coloring the landscape by each angle (Figure 2d) shows that different regions of the map capture distinct subunit–subunit geometries, and that moving vertically across the map corresponds to a change in tangential bending, including a flip of sign, i.e. a rotation in two different directions. This nicely shows that the map, which was generated from RMDs, i.e. features focusing on the intradimer interface, resolves global (re)­orientation of the two subunits.

Generally, the map shows that tubulin dimers sample a broad spectrum of curved conformations. This heterogeneity is consistent with the ability of microtubules to bend and twist in different directions while maintaining their function.^27^

### 2.2 Partial Memory of the Starting Structure Due to a Salt Bridge

We decided to more closely investigate the mentioned issue of incomplete convergence with regard to subunit–subunit orientation. Highlighting the areas of the conformational landscape initiated from different starting structures reveals that they overlap only partially (Figure 3a, with an overlap coefficient of 0.39). The bottom “tail” of the landscape consists of frames from the single anomalous GDP1 simulation after GDP dissociated from the nucleotide-binding pocket and these frames are not included in the purple density contour lines. For the remaining trajectories, the peach contour lines (representing 98%, 60%, and 30% density fractions) extend horizontally from left to right, while the purple contour lines extend vertically. This partial overlap suggests that the 3J6F- and 3J6E-initiated simulations explore conformational landscapes that do not fully intersect.

**Figure 3:**
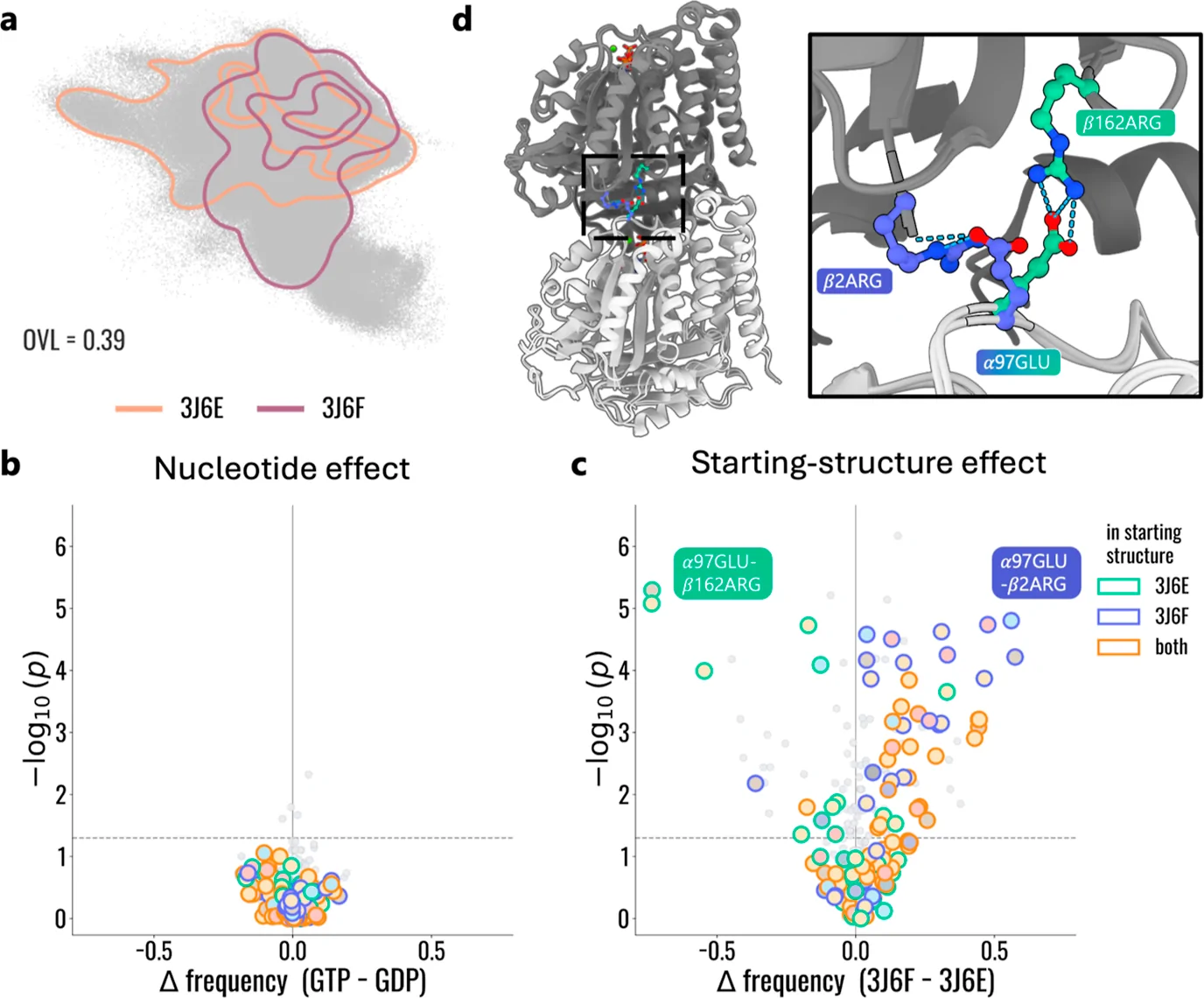
(a) Conformational landscape for the subsets of the data set initiated from the two starting structures (peach: 3J6E, purple: 3J6F), with density contour lines enclosing 98%, 60% and 30% of the density. Frames of GDP1 replica after GDP dissociated from the nucleotide-binding pocket are excluded from the density contours. OVL stands for the overlap coefficient and represents the fraction of density shared by the two subsets, where 1 would indicate identical distributions. Gray points show the projection of the entire data set. (b,c) Volcano plots of the effect of the nucleotide state (b) and of the starting structure (c) on the intradimer interface contacts during the first 10 ns of each simulation replica. Each point is a contact between an α- and a β-subunit residue. The *x*-axis is the difference in mean contact frequency between the two groups (GTP, *n* = 12 vs GDP, *n* = 12 in (b); 3J6F, *n* = 15 vs 3J6E, *n* = 15 in (c)), and the *y*-axis is the significance of that difference (two-sided Mann–Whitney *U* test, dashed line at *p* = 0.05). Points are outlined according to whether the contact is already present in a starting cryo-EM model (green: 3J6E only, blue: 3J6F only, orange: both). (d) The two superimposed starting models with a close-up of α97GLU reaching different partners: β162ARG in 3J6E (green) and β2ARG in 3J6F (blue).

To investigate the underlying reason for this divergence, we analyzed the early stages of the simulations, focusing on interactions at the intradimer interface. We used ProLIF^28^ to identify residues forming contacts during the first 10 ns of each of the 30 replicas. For every replica, each interaction was represented as an interaction frequency (the fraction of frames in which the contact was present). We then compared these interactions across nucleotide states (GTP: *n* = 12 vs GDP: *n* = 12) and starting structures (3J6F: *n* = 15 vs 3J6E: *n* = 15) using the difference in mean frequencies, displayed as volcano plots (Figure 3b,c). Each point in these plots represents a specific interaction between the α- and β-subunits: the *x*-axis resolves the change in frequency between the two groups (i.e., across nucleotide states in panel b and across starting structures in panel c), while the *y*-axis reflects the statistical significance across replicas. Consequently, points in the upper-right and upper-left corners represent interactions that vary both considerably and consistently under the respective change.

A comparison of Figure 3b (nucleotide effect) and Figure 3c (starting-structure effect) reveals different patterns. Most points in Figure 3b cluster near zero and fall below the significance threshold (*p* > 0.05). Conversely, changing the starting structure significantly affects the interface interactions. For instance, the most prominent structure-dependent contact is the α97GLU-β162ARG salt bridge (Δfrequency = −0.73), which forms frequently in 3J6E-initiated replicas but rarely in those started from 3J6F. This difference can be traced back to the specific interactions present in the initial cryo-EM models. Figure 3d depicts both starting structures and a close-up of α97GLU reaching out to different ARG partners: β162ARG in 3J6E (green) and β2ARG in 3J6F (blue). The α97GLU-β2ARG pair also appears in the upper-right corner of the volcano plot in Figure 3c, confirming its higher frequency in 3J6F-initiated simulations. Thus, the specific starting structure biases α97GLU to interact with one specific ARG over the other within the 10 ns time scale.

Why do these distinct salt bridges exist in the starting structures, and are they linked to the nucleotide state of the β-subunit? These starting structures were derived from a cryo-EM study of microtubule lattice patches^22^ with reported resolutions of 4.7 Å for 3J6E and 4.9 Å for 3J6F. At these resolutions, the experimental density does not resolve the exact position of side chains of residues such as ARG and GLU. Instead, RosettaCM was used to predict the final atomistic models. Ultimately, because these side-chain conformations were predicted by the RosettaCM energy function rather than being directly observed in the EM density, these salt bridges do not necessarily stem from nucleotide-induced differences in the original cryo-EM data.

In summary, we see that early interface contacts are affected considerably by the initial conformation of the dimer which should not automatically be ascribed to the identity of the bound nucleotide. This finding necessitates a cautious design and interpretation of subsequent analyses that aim toward addressing how the nucleotide state affects communication in the protein dimer. Thus, in all subsequent analyses we will keep track of the starting structure in addition to looking at the nucleotide state.

### 2.3 Clustering of the Landscape Presents Geometrically Homogeneous States

A more detailed characterization of the underlying conformational ensemble is possible after clustering the structures based on how the data points are distributed on the map. We use the Hierarchical Density-Based Spatial Clustering for Applications with Noise (HDBSCAN)^29^ clustering algorithm to identify density peaks in the two-dimensional projection. The main advantage of HDBSCAN is its ability to identify clusters with varying densities without prior assumptions about the number of clusters.^30^ In Figure 4a, the map is displayed according to the local density of data points, shown as filled contours on a logarithmic density scale. Darker shades of gray correspond to regions containing a larger number of simulation structures. We observe densely populated regions in the central area of the map, which corresponds to the largest amount of sampling. Clustering yields 14 clusters (denoted by indices as cluster 0—cluster 13; see Figure 4b, with cluster outlines also overlaid on the density map in Figure 4a). A first assessment of the quality of the clustering was obtained with the help of the pairwise Jensen-Shannon distances between the distributions of bending angles (Figure S5). There the within-cluster distances are low and between-cluster distances are higher, which confirms that the RMD landscape together with density-based clustering recovers geometrically coherent and distinct conformational states.

**Figure 4:**
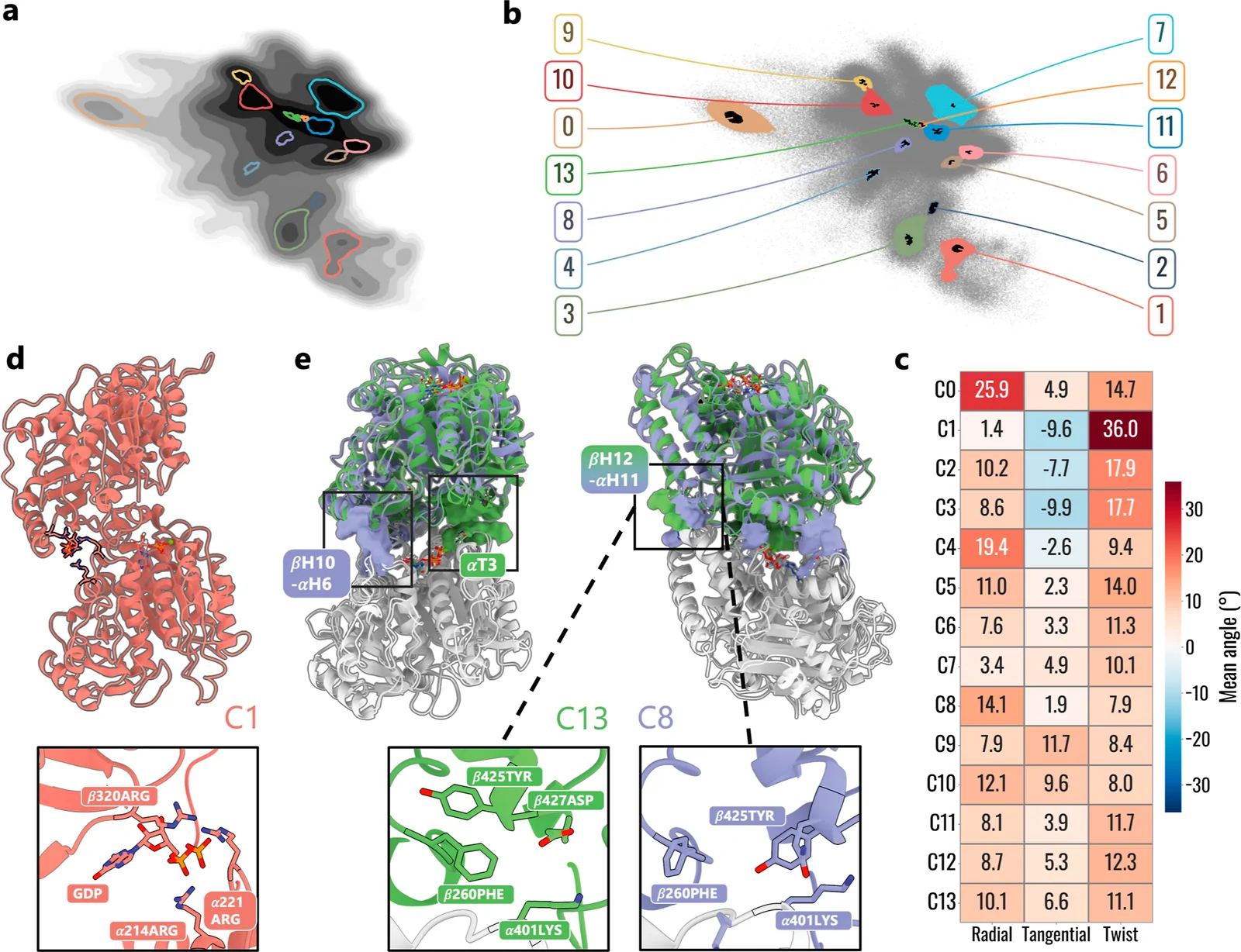
(a) Density of the EncoderMap projection displayed as filled contours on a logarithmic density scale (darker = more densely sampled). The colored lines are the outlines of the 14 HDBSCAN clusters, each drawn in its cluster color. (b) The same projection with the frames assigned by HDBSCAN to each cluster overlaid in the cluster color. Gray points are classified as noise, and the black points are the HDBSCAN exemplars used for the network analysis. (c) Mean value of the three bending angles (radial, tangential, twist) for each cluster. (d) Representative structure of cluster 1, with a close-up of the GDP that has dissociated from the β-subunit pocket and whose phosphates simultaneously coordinate β320ARG, α214ARG and α221ARG. (e) Representative structures of cluster 13 (green) and cluster 8 (purple), aligned on the α-subunit (white), in a lumen-facing view (left) and a side view (right). Throughout, orientations are described as viewed from the microtubule lattice (Figure S4). Surfaces mark residues that contact the opposite subunit more frequently in the respective cluster. The two close-ups show the shared contact zone.

In the following, general trends describing the clusters in the various regions of the map are described. Details regarding the cluster compositions and cluster sizes can be found in Figure S6. Generally, clusters in the low-density regions at the outer circumference of the map are populated mainly by one simulation replica, thus representing simulations which are trapped into peculiar states. For example, cluster 1 consists of structures that exhibit an unusually large mean twist angle value of 36° (Figure 4c). As mentioned above this “tail” area of the map stems from the GDP1 simulation and cluster 1 contains frames where the GDP, which had dissociated from the β-subunit pocket, found interaction partners on the side of the protein. Its phosphates simultaneously coordinate 3 arginines (Figure 4d) locking the subunits into the strongly twisted geometry. Thus, the clustering analysis shows that the peculiar overall behavior of the GDP1 simulation is not due to the dissociation per se, but rather to the very specific interactions of the free nucleotide after dissociation in this simulation, i.e. it should not be overinterpreted. Cluster 0, visited mainly by GDP-swapped simulations, shows high radial bending angle values (with a mean radial bending of 25.9° for this cluster). This conformation is more strongly bent than any experimentally resolved intradimer structure. Across a range of nonmicrotubular tubulin structures, the reported intradimer curvature spans from approximately 9° up to a maximum of 18.2°,^31,32^ depending on the binding partner. Even allowing for slightly different angle definitions, this places cluster 0 in a bending regime beyond the one captured by current experimental structures, consistent with it being a rarely visited, metastable state.

Setting these rare clusters aside, we focused on the clusters in the densely populated central region of the landscape, which were visited by many replicas and by simulations across all conditions, i.e. both nucleotide states and from both starting structures (Figure S6). These clusters differ in their specific combination of bending angles, that is in the relative orientation of the two subunits (Figure 4c). Beyond their bending angles, the clusters differ in the contacts they form across the longitudinal α–β interface, which we characterized for every cluster with ProLIF analysis (see Figure S7 for a list of interface contacts of all clusters and an illustration). Viewed from the α-tubulin side, the interface separates into an invariant hydrophobic core and a set of cluster-specific contacts. In all clusters the C-terminal helices of α-tubulin (αH11 and αH12) stay in contact with β-tubulin through a few invariant hydrophobic residues, although the polar contacts within this region are redistributed from cluster to cluster. The other contact zones, like the αT3 and αT5 loops and the αH6–αH7 region, are engaged only in a subset of clusters.

In the following we focus on two of these clusters for which this behavior can be observed in more detail. Figure 4e shows representative structures of cluster 8 (purple) and cluster 13 (green), aligned on the α-subunit. The two clusters adopt distinct subunit orientations: cluster 8 structures are more radially bent with a smaller tangential component, whereas cluster 13 structures are less radially bent but carry a larger, more positive tangential bending angle (Figure 4c,e).

This reorientation can be traced to the interface between the two subunits. The per-cluster ProLIF analysis of the contacts between subunits (Figure S8) reveals a redistribution of interface salt bridges between the two clusters: two interface zones differ completely, and a third is engaged differently. In cluster 13, a set of salt bridges forms on one lateral side of the dimer around the αT3 loop (residues with green surface, lumen-facing view in Figure 4e), where α97GLU is the most frequently contacting residue. In cluster 8, salt bridges instead form in a different patch, at the αH6−βH10 interface (residues with blue surface, lumen-facing view in Figure 4e). Because the cluster 13 patch lies on a lateral side of the dimer, its engagement is consistent with the larger tangential bending of this cluster. The third zone, namely the contact between the C-terminal helices, αH11-βH12 (Figure 4e, rotated side view and close-ups), is shared by both clusters but differs in terms of the exact interactions. In cluster 13, α401LYS engages β427ASP and β260PHE, leaving β425TYR free to π-stack with β260PHE. The presence of this stack correlates with the larger dimer twist. In cluster 8, α401LYS instead engages β425TYR, drawing it toward the α-subunit and away from β260PHE, so the aromatic stack is broken and β260PHE can flip into the trans rotamer (Figure 4e).

Taken together, the clustering shows that the densely sampled central region of the landscape is not a single homogeneous basin but a set of conformational substates that differ mainly in the relative orientation of the two subunits. These orientational differences go along with the pattern of contacts across the longitudinal interface.

### 2.4 Betweenness Centralities Can Capture Nucleotide Effect

So far, neither the conformational landscape nor the bending angles revealed a clear difference between GDP- and GTP-tubulin. Still, the absence of a single γ-phosphate is what ultimately makes the difference between the microtubules’ phases of growth and shrinkage. This suggests the presence of an allosteric network within the dimer that can transmit nucleotide-state information within and across the domains. We therefore asked whether the nucleotide state is encoded in the way its residues communicate.

MD simulations in combination with graph analysis provide an effective framework to uncover such long-range couplings.^33^ Therefore, to investigate communication within the dimer, we employed dynamic network analysis. The main idea here is to represent the protein system using a graph. The residues become nodes of the graph which are connected with edges, that have associated correlation coefficients, in the present work mutual information coefficients (more details on the graph construction see Methods section). Graph-theoretical metrics then allow the identification of central nodes and communication pathways.

Two features of our simulations data set motivate the way how the networks are constructed. First, because the simulation set contains multiple conformational substates, averaging over all the trajectories during network construction would mix these different substates.^15^ As a result, one would get either misleading results or a “diluted” signal that is hard to decode. Second, the ensemble is not fully equilibrated. Alongside the central area of the conformational landscapes with well-sampled substates, we observe some substates that are visited by only a single replica. To deal with these challenges, we build a network per cluster, and within each cluster per condition, instead of one network for the whole data set. The central clusters are well suited for this, since several of them are repeatedly visited by all four nucleotide × starting-structure conditions (Figure S9a). We weight the edges by mutual information, which adds information about how residues covary and lets us trace more subtle effects such as the influence of the nucleotide state.

In HDBSCAN, each cluster is assigned a list of exemplars, defined as structures that remain within the cluster over the broadest range of density thresholds in the condensed tree. In practice, exemplars correspond to the most prevalent and therefore most representative conformations of each substate, making them a natural choice for constructing cluster- and condition-resolved networks. On the conformational landscape in Figure 4b the location of the exemplars is shown as black dots. For that reason, using cluster exemplars ensures each graph reflects the representative geometry of that substate while avoiding heavy computational costs and noise from processing a large number of individual frames. We confirmed that the exemplars reflect the composition of their clusters with respect to starting structure and nucleotide state, and that they reproduce the cluster geometry, as shown by their bending angles distributions (Figure S9b). Contact probabilities and mutual information were computed from the exemplar frames, and the resulting averaged matrices were used to construct a single weighted network per cluster per condition. Graph metrics were computed per cluster-condition-graph.

At first, we looked at the betweenness centrality (BC) as a global network metric. It quantifies how frequently a node is included in the pairwise shortest paths between other nodes. A residue with high BC is thought to control the communication flow within the network. In the context of proteins, it was shown that betweenness centralities can pinpoint biologically relevant residues.^34^

Most residues show low betweenness (Figure S10), meaning they do not strongly influence how information propagates through the structure. This is expected and reflects the general robustness of protein networks: communication can proceed through many alternative routes even if individual low-betweenness residues are perturbed. In contrast, a small subset of residues exhibits high betweenness. These residues form communication bottlenecks and represent points of fragility in the network:^35^ because many shortest paths depend on them, perturbing such residues is likely to disrupt information flow and impair the protein’s function. Comparing the raw betweenness across all cluster–condition networks (Figure S10), the high-betweenness residues reoccur at the same positions almost independently of the individual network. They concentrate in a few broad regions at the intradimer interface. On the α-side the T2, T3 and T5 loops, which coordinate the nonexchangeable GTP of the α-subunit, and on the β-side helix H8 and its flanking loops. The same secondary-structure elements therefore play the bottleneck role in the communication in every cluster and condition. What changes from one network to the other is the role of individual residues within these broader structural elements.

Although the raw betweenness-centrality values reveal the principal communication bottlenecks, they do not separate the contribution of the nucleotide state from that of the starting structure. To disentangle these effects we leverage the design of our networks and average the betweenness over the two starting structures. This analysis requires clusters whose exemplars contain networks for all four conditions, which we can then treat as 2 × 2 factorial design (nucleotide ∈ {GDP, GTP} × starting structure ∈ {3J6E, 3J6F}). The two clusters 8 and 13 which had been described earlier are particularly well-suited for a statistically sound analysis of the four conditions (details regarding the number of exemplars across the four conditions for these clusters are given in Supporting Information Section S9). For each cluster we built four independent mutual information-weighted networks and computed the BC of every residue in each of them. We then decomposed the four BC values of each residue into three effects: a nucleotide effect *E*_nuc_ (the change in BC between GTP and GDP, averaged over the two starting structures), a structure effect *E*_str_ (the change between 3J6E and 3J6F, averaged over the two nucleotides), and an interaction term *E*_int_. The interaction term is nonzero when a residue responds to the nucleotide only for one of the starting structures, for example when the betweenness is higher under GTP only in the simulations started from 3J6E (see Methods for more details). For such elements the nucleotide effect is not separable from the starting-structure effect, so we do not interpret them and restrict our discussion to the nucleotide-clean parts of the network. A residue with a large |*E*_nuc_| and a small |*E*_int_| is a hub that responds to a given nucleotide independently of the starting structure.

In order to show for which residues the BC responds to the particular effect, we visualized each residue as a point in a (|*E*_nuc_|, |*E*_str_|) scatter plot, with the marker size proportional to 1/|*E*_int_|, so that residues that respond cleanly to a single factor are displayed large and lie close to one of the axes (Figure 5). Residues whose effect is above a threshold (dashed arc) are colored by their dominant factor and its direction: nucleotide-clean (green if BC is higher under GTP, blue if higher under GDP), structure-clean (peach if higher in 3J6E, purple if higher in 3J6F), or interaction-dominated (dark gray).

**Figure 5:**
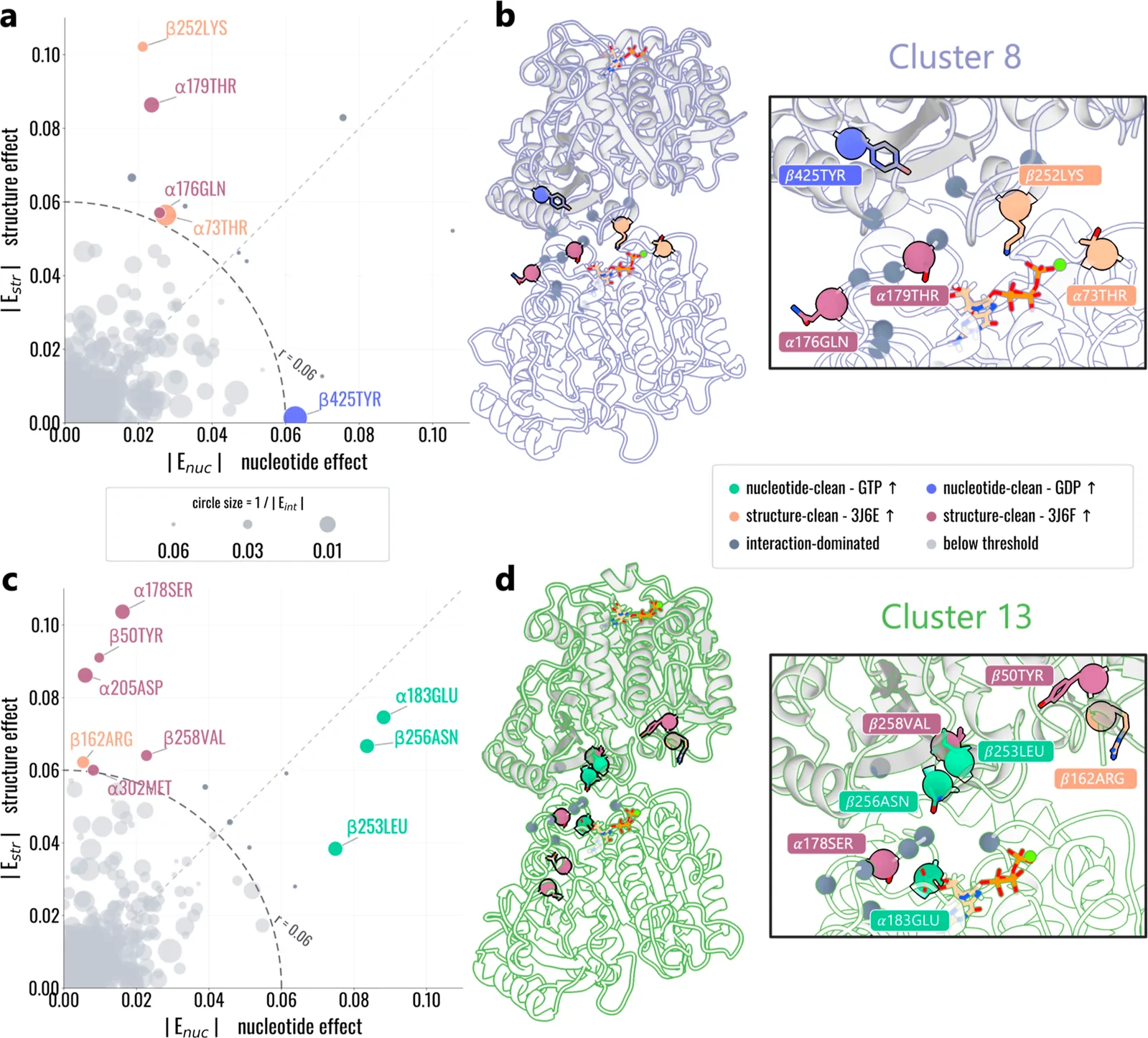
Factorial decomposition of residue BC for cluster 8 (a,b) and cluster 13 (c,d). (a,c) Each residue is plotted using the magnitude of its nucleotide effect (|*E*_nuc_|, *x*-axis) and structure effect (|*E*_str_|, *y*-axis). The marker size is proportional to 1/|*E*_int_|. Residues beyond the dashed arc are colored by their prevailing factor and its direction: nucleotide-clean (green: higher betweenness under GTP, blue: under GDP), structure-clean (peach: higher in 3J6E, purple: higher in 3J6F), or interaction-dominated (dark gray). The radius *r* = 0.06 corresponds to the 99.sixth (cluster 8) and 99.second (cluster 13) percentile of a permutation null distribution. (b,d) Residues from the scatter plot mapped onto the representative dimer structure of cluster 8 (purple) and cluster 13 (green). The boxed close-ups show the intradimer interface. The color legend is shared by all panels.

Most of the residues with a high effect localize at the interface between the two subunits, hinting that the intradimer interface is important for allosteric communication and suggesting that this is where the communication within the dimer is most affected by the nucleotide and the starting structure. The residues that stand out differ between the two clusters.

In cluster 8 one residue is nucleotide-clean, β425TYR, whose betweenness is higher when the nucleotide is GDP. β425TYR already appeared in the contact analysis above, where its π-stacking with β260PHE was less persistent in cluster 8 than in cluster 13. Here the same residue stands out as the one whose network role responds to the nucleotide. β425TYR is located on the outer C-terminal (βH12 helix) surface. A GDP-specific increase of its centrality would pose a possible way for the nucleotide state to be communicated to the surface. This might be interesting because several microtubule-associated proteins and molecular motors show a selectivity for the nucleotide state of the dimers in the microtubule lattice. For example, tau protein, that acts as a microtubule stabilizer in neurons, preferentially binds to the GDP-bound microtubule lattice.^36^ The H12 helix that contains β425TYR is a known region for interactions with the microtubule-binding domains of these proteins, such as the R2 microtubule-binding repeat in the case of tau.^37^ Together, these observations suggest that nucleotide-dependent changes in the communication network may propagate to the outer surface of the dimer, potentially modulating the binding of state-sensitive partners.

In cluster 13 three residues are nucleotide-clean, α183GLU, β256ASN and β253LEU, all with higher betweenness when the nucleotide is GTP. They are located along the longitudinal intradimer interface on both sides: α183GLU at the αT5 loop, as well as β253LEU and β256ASN on helix H8 of β-tubulin, that interact across the longitudinal interface with the GTP molecule that is bound in the α-subunit. Interestingly, between S9 and H8 in the β-subunit, a binding site for the microtubule binding drug colchicine is located.^38^ It disrupts the microtubule polymerization cycle due to its position at the intradimer interface. Based on the position, it is thought that only bent tubulin conformations can accommodate the molecule and after colchicine binding the dimer is incompatible with a straight lattice conformation.^39^ Colchicine’s binding site therefore overlaps with the interface residues identified as nucleotide-clean here. This observed overlap raises the possibility that colchicine could influence intradimer communication, but more direct investigation would be required to conclusively address this question.

Taken together, the betweenness analysis reveals a nucleotide effect for specific conformational states of the dimer that is carried by a few residues at the intradimer interface. Even though the effect is small, it is potentially interesting from a mechanistic perspective since it points to residues or interaction sites that have previously been identified experimentally.

### 2.5 Shortest Path Analysis Indicates Allosteric Communication Across Dimer Interface

To further characterize information transfer in the tubulin dimer, we employed a directional metric from graph theory - shortest path analysis. Rather than looking globally at the network, one specifies two regions of “source” and “sink” nodes based on prior knowledge about the system and investigates the communication between those regions.

We placed the source nodes on the upper (plus-end) surface of the β-subunit where the binding of the exchangeable nucleotide occurs and the sink nodes on the lower (minus-end) surface of the α-subunit (see Figure S11 for an illustration and Table S1 for a list of source and sink residues). In the microtubule lattice these are the residues that contact the longitudinally adjacent heterodimers, so the paths between them have to cross the intradimer interface and describe the longitudinal communication through the dimer.

We calculated the paths between the source and the sink regions for the two clusters with the same 2 × 2 (nucleotide × starting structure) factorial design that had been used for the betweenness analysis above. Both for cluster 8 and cluster 13 we computed the paths separately for the four conditions and recorded two quantities: how often each residue lies on a path (a node frequency), and how often a given residue–residue connection is used in a path (an edge frequency). Again, we decomposed both node- and edge frequency into a nucleotide-, a structure-, and an interaction effect, as for the betweenness. The result is shown in Figure 6 where the longitudinal communication in the dimer is illustrated for clusters 8 and 13: residues are indicated as balls with color and size according to (nucleotide-clean) node frequency, and residue–residue connections as sticks with color and thickness according to (nucleotide-clean) edge frequency. To keep the structures readable we show only the most frequently used route elements and highlight the nucleotide-clean elements, therefore routes can appear fragmented.

**Figure 6:**
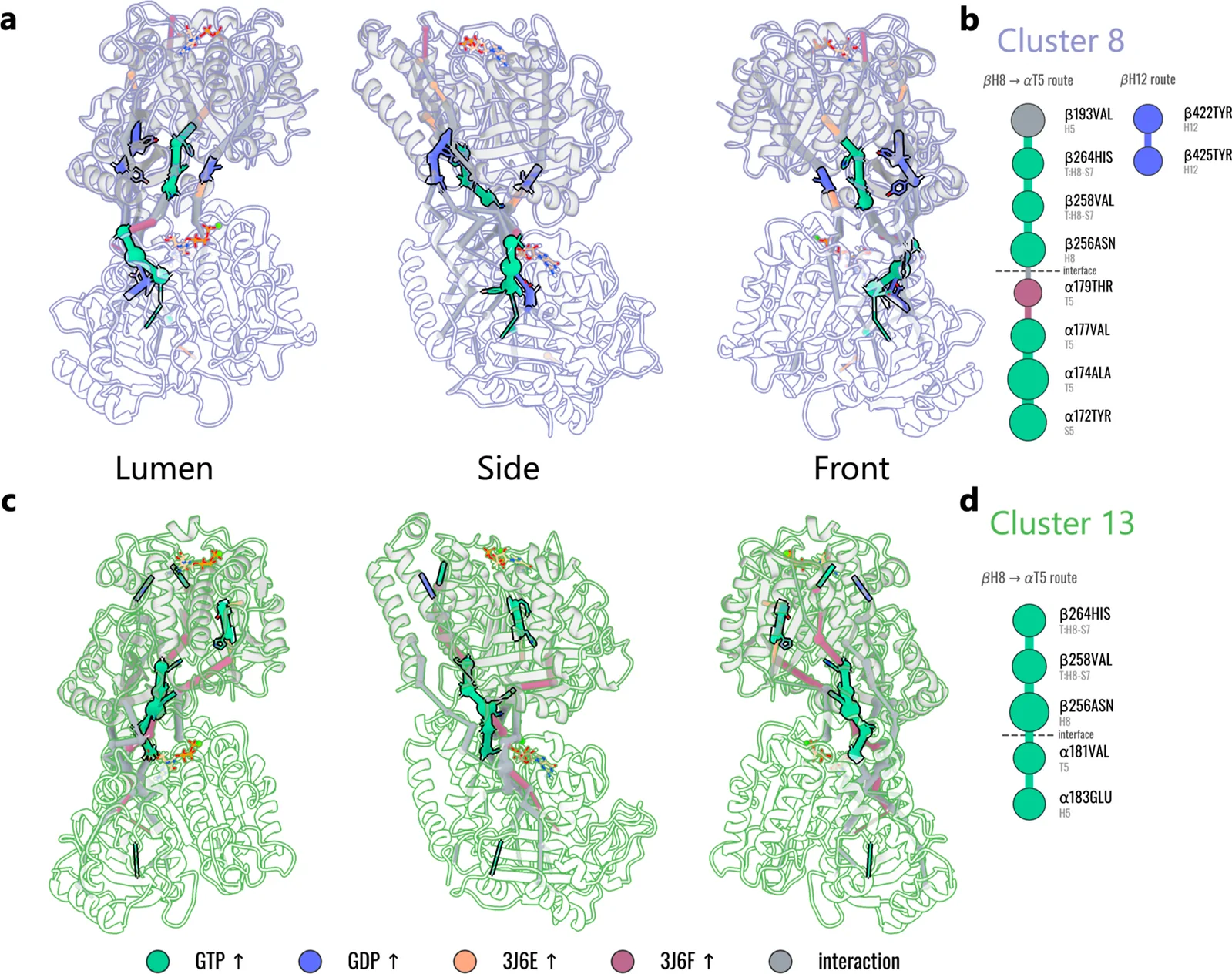
Longitudinal communication paths in cluster 8 (a,b) and cluster 13 (c,d). (a,c) The path network mapped onto the representative dimer structure (cluster 8: purple, cluster 13: green), in three orientations (lumen-facing, side and front). Balls indicate the most-used residues (above the 90th percentile of node frequency), with radius proportional to node frequency. Pseudobonds show the most-used edges, with thickness proportional to edge frequency. The top nucleotide-clean residues are additionally shown in a stick representation. The color throughout encodes the dominant factorial effect similar to Figure 5b,d Simplified diagrams of the selected routes that carry a clean nucleotide signal.

In both clusters the source–sink paths are funneled through the interface regions that had previously been identified in the betweenness analysis: helix H8 and its H8–S7 flanking loop on the β-subunit and the T5 loop on the α-subunit.

In cluster 8 the paths follow an almost continuous longitudinal route from βH8 to the αT5 loop (Figure 6a,b) that responds to GTP. The β-side segment along H8 and its H8–S7 loop (β264HIS → β258VAL → β256ASN) and the α-side segment along the T5 loop and S5 strand (α177VAL → α174ALA → α172TYR) are both GTP-clean. The other clean nucleotide signal in Cluster 8 is located on the outer C-terminal surface of β-tubulin: the β422TYR → β425TYR edge on helix H12 responds to GDP (Figure 6b).

In cluster 13 the same general βH8−αT5 route is present and also responds to GTP being bound in the β-subunit (Figure 6c,d). It differs from the route in cluster 8 in the specific residues engaged in the αT5 loop, namely α181VAL → α183GLU. We thus observe a conserved route βH8−αT5 that is activated by GTP in both substates.

The residues that make up these routes can be viewed in the context of genetic data on tubulin, where even minor sequence changes cause severe neurodevelopmental disorders.^40^ To test more directly whether our communication residues are functionally important, we compared them with a recent high-throughput deep mutational scan of human α-tubulin,^41^ in which every single-residue substitution was scored for its effect on microtubule assembly (the assembly score). A low assembly score marks positions where substitutions harm assembly. The α-side residues of our route are enriched among these positions. Most notably, α183GLU, the residue that carries the clean GTP signal across the interface in cluster 13, ranks among the most assembly sensitive residues (eighth percentile) of α-tubulin. The interface-crossing residue α181VAL and the S5 hub α172TYR are also strongly assembly sensitive. The fact that our network analysis and an independent functional screen identify the same residues supports the biological relevance of the longitudinal route we identify. More generally, residues that lie on allosteric communication paths are regarded as candidate sites for pharmacological targeting, since they offer a way to modulate protein function from outside the orthosteric site.^11,14^ In this regard, the nucleotide-responsive hubs identified here in combination with the structural representatives from the simulations provide sites for targeted mutagenesis and for the design of ligands aimed at modulating communication within the dimer.

In the canonical allosteric model of tubulin dynamics, the nucleotide controls the curvature of the dimer. GTP-tubulin is straight and able to polymerize, while GDP-tubulin is bent and therefore unable to be incorporated into the microtubule lattice. Contrary to that, subsequent studies have shown that the tubulin dimer is predominantly bent in solution and GDP-tubulin itself is able to polymerize at high concentrations via minus-end growth.^42^ It has been discussed that this points to a lattice-coupled structural switch, in which GTP does not merely “straighten” tubulin but instead biases an underlying conformational transition that is catalyzed by polymerization itself.^3^ In this view, the nucleotide state becomes one allosteric effector among many that modulate how tubulin responds to its structural context. Our work adds an intradimer perspective to this framework. Within our set of simulations we do not observe that the nucleotide reroutes the longitudinal communication. Rather, what we can observe is the preferential use of specific edges for the longitudinal communication across the interface of the dimer.

Sequence-wise, tubulin experiences high evolutionary constraints, especially at the intra- and interdimer interfaces, to preserve the key process of microtubule polymerization.^43^ Different organisms and cells within them can control microtubule dynamics through the expression of various tubulin isoforms,^44^ the recruitment of different microtubule-associated proteins, and various post-translational modifications.^45^ The allosteric network within the dimer found in our study provides an additional control layer to tune microtubule dynamics, one among several that shape behavior at the scale of the lattice.

Limitations should be kept in mind when interpreting results of this study. As we have shown, the relative reorientation of the two subunits is a slowly varying degree of freedom, and on the time scales accessible here the simulations retain a partial memory of their starting structure. We addressed this explicitly with a factorial design that averages over the two starting structures. In turn, the interaction-dominated results (where the analysis cannot separate the nucleotide from the starting-structure contribution) can not be interpreted head-on and are not further discussed here due to the nature of the data set at hand. Overcoming this would require more exhaustive sampling of the subunit–subunit orientation, for example through enhanced-sampling or coarse-grained simulations and a larger number of replicas. We also observed nucleotide dissociation from the β-tubulin binding pocket in the simulation data set. Here, too, sampling limitations apply, and obtaining a quantitative description of the dissociation process would require dedicated approaches such as transition path sampling. Such approaches require a diverse set of starting conformations, which the cluster representatives identified in this work could provide.

## 3 Conclusions

In this work, we set out to determine how the nucleotide in the β-tubulin subunit affects the intrinsic dynamics of the αβ-tubulin dimer and whether it changes communication patterns across the longitudinal interface between the two subunits. Using extensive all-atom MD simulations of the tubulin dimer with GTP or GDP in the nucleotide-binding pocket of the β-subunit, we first established the global landscape of subunit–subunit orientations. For that we utilized RMDs as feature vectors. This internal coordinate set proved well-suited for resolving interface changes between the protein subunits. By applying EncoderMap dimensionality reduction to these features, we obtained a low-dimensional conformational landscape. We found that the dimers relax from their straight starting structures into a broad ensemble of bent conformations. The identity of the bound nucleotide did not cleanly separate GDP- from GTP-tubulin and we observed a partial memory of the starting structure. We traced part of this starting-structure memory to a salt bridge of α97GLU that differs between the two cryo-EM starting models. Building on this global structural characterization, we separated the conformationally heterogeneous landscape into substates with density-based clustering and used these substates for the network construction. The conformational heterogeneity of the data set and the residual influence of the starting structure motivated a substate- and condition-resolved network analysis. We therefore constructed networks separately for individual conformational clusters and, within each cluster, for each nucleotide × starting-structure combination. BC analysis showed that residues whose communication role responds to the nucleotide are concentrated at the longitudinal intradimer interface. Directional shortest-path analysis provided a complementary picture showing that longitudinal communication consistently passes through a conserved interface region βH8−αT5. More broadly, our results demonstrate that nucleotide-dependent allosteric effects can still be resolved despite incomplete conformational convergence. Moreover, combining dimensionality reduction with substate- and condition-resolved network analysis provides a general strategy for disentangling subtle allosteric signals from heterogeneous MD simulations.

## 4 Methods

### 4.1 MD Simulations

Initial models for the tubulin dimers were obtained from PDB IDs 3J6E (GMPCPP) and 3J6F (GDP) by extracting the central dimer (chains A and B in the original PDBs). GMPCPP was converted into GTP by replacing the carbon atom between α- and β-phosphate with an oxygen atom. The resulting GTP molecule was parametrized using the corresponding CHARMM36m force field parameters. The missing loop in the α-subunit (residues 39–48) was modeled in for structure consistency using MODELLER version 10.4.^46^ In addition, we prepared swapped sets of simulations and apo sets of simulations. In the swapped system the nucleotide in the β-subunit was interchanged from the experimentally obtained structures, i.e, GDP was placed into the β-subunit nucleotide pocket of 3J6E and GTP was placed in the pocket of 3J6F. In the apo simulations the nucleotide was removed from the β-subunit of both of the initial experimental protein conformations. Mg^2+^ was included at every nucleotide-binding site occupied by GTP in line with the cryo-EM starting model. All simulations were performed with the GROMACS 2022.4 simulation package^47,48^ and CHARMM36m force field.^49^ Initial conformations for simulation were obtained by placing a dimer in a dodecahedron box such that the protein had a minimum distance of 15 Å to all box edges. The box was filled with CHARMM-modified TIP3P water molecules.^50^ No background salt was added, Na^+^ was introduced to neutralize the net charge of the solute. Initial structures were relaxed through energy minimization using the steepest descent algorithm. The systems were then equilibrated in two steps: (1) a 100 ps run under constant temperature (*NVT*) with position-restrained backbone and side chain atoms; (2) a 100 ps run under constant temperature and pressure (*NPT*) also with position-restrained backbone and side chain atoms. The production simulation runs were carried out in the *NPT* ensemble at 300 K and 1 bar using the Nosé–Hoover thermostat^51^ and the Parrinello–Rahman barostat,^52^ respectively. The default md (leapfrog) integrator was used with a 2 fs time step. Neighbor searching to generate a pair list was done using the Verlet cutoff scheme. Short-range electrostatic interactions and van der Waals interactions were calculated with a 1.2 nm cutoff. To treat the long-range electrostatics, the smooth particle-mesh Ewald method was used.^53^

### 4.2 Intradimer Interface Analysis

Interactions across the α–β interface were detected with ProLIF.^28^ ProLIF was used both for early dynamics assessment and for interaction identification in the exemplar frames of the clusters. For the early dynamics analysis, interaction fingerprints were computed for the first 10 ns of each replica. The per-replica interaction frequencies were grouped by nucleotide state (GTP and GDP) and by starting structure (3J6E and 3J6F), and each interaction was compared between groups using a two-sided Mann–Whitney *U* test. The results are presented as volcano plots showing the difference in mean interaction frequency versus −log_10_ *p* (significance threshold *p* = 0.05).

### 4.3 Intradimer Bending Angles

Analysis of the bending motion of one subunit relative to another was similar to the established procedure.^26^ Microtubule fragment (PDB ID 3J6F) was used to create a microtubule-bound coordinate system in the following way: for every time frame of the dimer simulation, the terminal domains (helices and strands within the N- and C-terminal domains) of the α-subunit were aligned to the microtubule fragment, creating *xyz* unit vectors, the other copy of the microtubule fragment was then aligned to the β-subunit, making the second *XYZ* coordinate system. To determine the Euler angles needed to convert one coordinate system to the other a rotation matrix was calculated. The Euler angles were extracted according to the *xyz* extrinsic method, giving rise to the following angles: θ for rotation around *x* axis representing the radial bending, ϕ for rotation around *y* axis representing the tangential bending and δ for rotation around *z* axis representing the twisting motion of one subunit relative to another (see Figure S4 for an illustration).

### 4.4 Dimensionality Reduction and Clustering

RMDs between two subunits were chosen to describe the relative subunit–subunit orientation. They were calculated with the GROMACS mindist module in the following way: for every residue in one subunit, the minimum distance to the atoms in the other subunit is calculated and vice versa. Therefore, every time frame is described by a vector of 864 distance values (438 residues in the α-subunit and 426 residues in the β-subunit). The simulations were analyzed with a time step of 100 ps. To obtain a low-dimensional representation of the conformational landscape of the tubulin dimer from the resulting 864-dimensional data set EncoderMap^54,55^ was used. Based on the high-dimensional distance distribution of RMD from the 10 μs simulations set, the sigmoid function parameters σ_*h*_ = 8, *a*_*h*_ = 8, *b*_*h*_ = 5, σ_*l*_ = 1, *a*_*l*_ = 2, *b*_*l*_ = 5 were chosen. A comparison with two alternative dimensionality reduction techniques, namely principal component analysis as a linear method and UMAP,^56^ as another nonlinear technique, is provided in Figure S12. Clustering of the two-dimensional projection was performed with HDBSCAN,^29^ where parameters were chosen to limit clustering to denser regions of the map (specific values used were minimum cluster size = 1000 and minimum samples = 1000). The algorithm extracted 1000 exemplars per cluster, which were subsequently used for the network construction. For the projection and clustering of the data all 30 simulation replicas were used. This includes the GDP1 replica, in which GDP leaves the β-tubulin pocket after ≈5.2 μs. Of the postdissociation frames, 75.5% of the GDP1 replica are classified as noise, and the clustered remainder falls almost entirely into cluster 1 (8562 of its 8630 frames) and cluster 3 (3075 of its 22,688 frames). Excluding these frames would therefore remove cluster 1 and only very slightly alter the composition of the other clusters.

### 4.5 Dynamic Network Analysis

To characterize allosteric signal transduction network analysis was used. To separate the nucleotide effect from the starting-structure effect, networks were built per cluster per condition. Within a cluster the exemplar frames were split into four conditions (nucleotide ∈ {GDP, GTP} × starting structure ∈ {3J6E, 3J6F}), and one network was constructed for each condition. To define the edges the shortest distance between heavy atoms of the nodes was calculated and two nodes were considered in contact if their distance was shorter than 4.5 Å and they were in contact in 75% of exemplar frames. Following the study by Sethi et al.^12^ the edges between the nearest neighbors were removed in order to avoid the emergence of trivial suboptimal paths during path analysis. The edges of the resulting graph were weighted with *w*_*ij*_ = −log­(*C*_*ij*_) using the mutual information as a correlation metric. Mutual information includes nonlinear contributions to the correlation.^57^ It was calculated with NetSci library following the “local alignment” approach.^58^ The construction of the graphs and the following network analysis were performed with the python package NetworkX.^59^ BC was used as a global metric of network connectivity. It is the number of shortest paths that go through the node which reflects its role in the communication within the network. The shortest path between two nodes is the path where the sum of the weights *w*_*ij*_ of the constituent edges is minimized and in our case the sum of correlation scores *C*_*ij*_ is maximized. BC was calculated for each of the four conditions in the clusters that contained representatives from all four conditions. For each residue within a given cluster, the four BC values corresponding to the four conditions were decomposed into a nucleotide effect (*E*_nuc_), a structure effect (*E*_str_), and an interaction term (*E*_int_) as follows

1 $$E_{nuc}=\frac{1}{2}\left[\left(BC_{GTP,3J6E}+BC_{GTP,3J6F}\right)-\left(BC_{GDP,3J6E}+BC_{GDP,3J6F}\right)\right]$$

2 $$E_{str}=\frac{1}{2}\left[\left(BC_{GDP,3J6E}+BC_{GTP,3J6E}\right)-\left(BC_{GDP,3J6F}+BC_{GTP,3J6F}\right)\right]$$

3 $$E_{\mathrm{int}}=\frac{1}{2}\left[\left(BC_{GTP,3J6E}-BC_{GTP,3J6F}\right)-\left(BC_{GDP,3J6E}-BC_{GDP,3J6F}\right)\right]$$

The resulting values of *E*_nuc_ and *E*_str_ were used to construct a scatter plot. Residues satisfying $r=\sqrt{E_{nuc}^{2}+E_{str}^{2}}\geq 0.06$ were visually highlighted. To obtain the null distribution of *r* for one cluster, we randomly assigned condition labels to the exemplar frames while preserving the original condition sizes. We used 200 permutations per cluster. Residues with a small interaction term (|*E*_int_| ≤ 0.03 (half the highlight radius)) were further classified as nucleotide-clean or structure-clean, and interaction-dominated otherwise (|*E*_int_| > 0.03).

For the directional path analysis, source nodes were placed on the plus-end surface of the β-subunit and sink nodes on the minus-end surface of the α-subunit—the residues that form the longitudinal contacts with the adjacent heterodimers in the microtubule lattice. For the exact residue list refer to the Supporting Information Section S11. Residue numbering throughout the paper follows the consecutive UniProtKB numbering without any gaps for β-tubulin (Uniprot ID: P02554), which were introduced by Löwe et al.^60^

## Supplementary Material



## Data Availability

The simulation data set underlying this study and example Python notebooks for the analysis are available in the research data repository KonDATA at the following link: https://doi.org/10.48606/hxx5n11uwxmt9gg4.

## Notes

Supporting Information includes the structure of the tubulin dimer with secondary structure elements depicted, time traces of individual trajectories mapped onto the EncoderMap projection, backbone RMSDs for all replicas, additional details on the calculation of bending angles, supplementary clustering statistics, raw betweenness centralities, a list of source and sink residues, and alternative dimensionality reduction techniques applied to the data (PDF)
