# Supplementary material for: Allosteric Communication in the Tubulin Dimer Revealed through Molecular Dynamics and Network Analysis
Source: ACS Omega. 2026 Oct 11;11(42):65580–93. doi: 10.1021/acsomega.6c08528 (PMC13718205; doi:10.1021/acsomega.6c08528)
Supplement: Supplementary file 1 [file ao6c08528_si_001.pdf]

SUPPORTING INFORMATION: Allosteric Communication in the Tubulin Dimer revealed through Molecular Dynamics and Network Analysis

Polina Teterina* and Christine Peter*

Department of Chemistry, University of Konstanz, Universitätsstraße 10, Konstanz 78457, Germany

E-mail: polina.teterina@uni-konstanz.de; christine.peter@uni-konstanz.de

SI Selected set of secondary structure elements

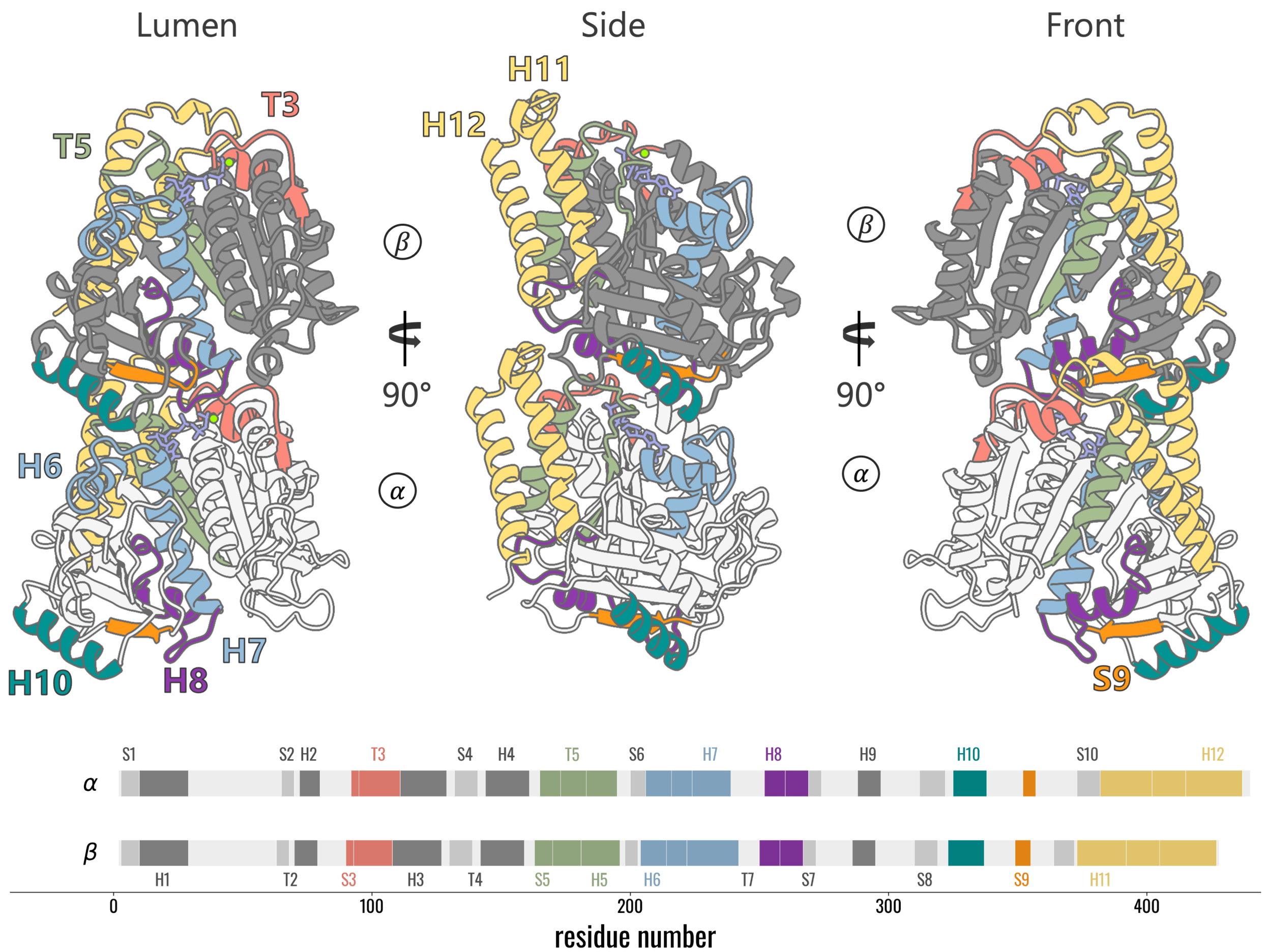

Figure S1: Illustration of the tubulin dimer and visualization of the secondary-structure elements referenced throughout the text. **Top:** the $\alpha\beta$-tubulin dimer (β on top, α on the bottom) in three orientations (lumen-facing, side and front). In both, the highlighted elements are colored as in the sequence below. Because α- and β-tubulin share the same fold, each element is colored identically in the two subunits.

SII Replica progress

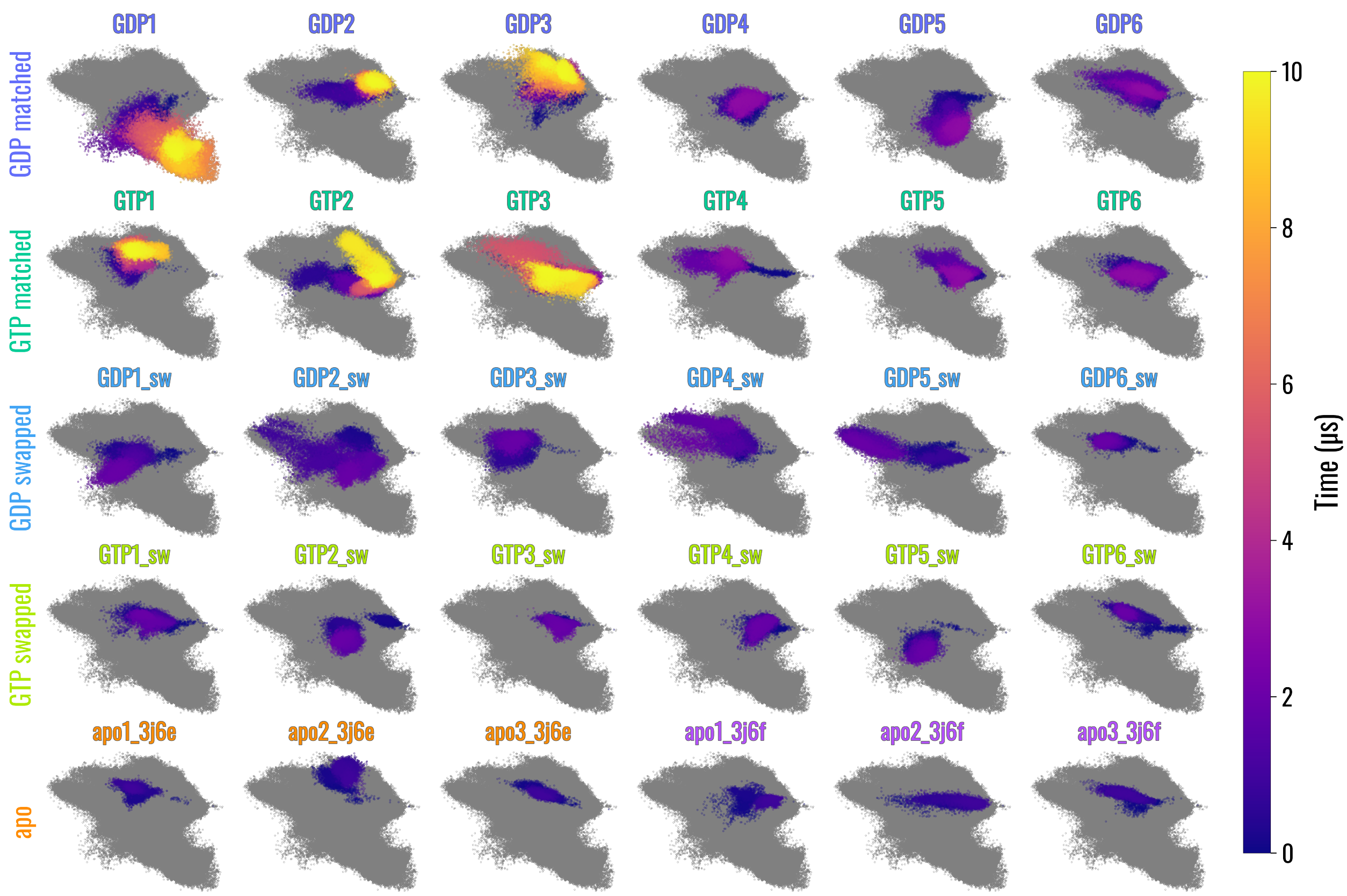

Figure S2: Time evolution of the individual simulation replicas on the global conformational landscape. Each panel shows one replica. Rows group the conditions (GDP and GTP matched, GDP and GTP swapped, and apo) and columns the replicas within each condition. Gray points show the projection of the whole dataset (the same landscape as in Figure 1**c** of main manuscript). The colored points are the frames of that replica, colored by simulation time.

SIII Backbone RMSD for all replicas

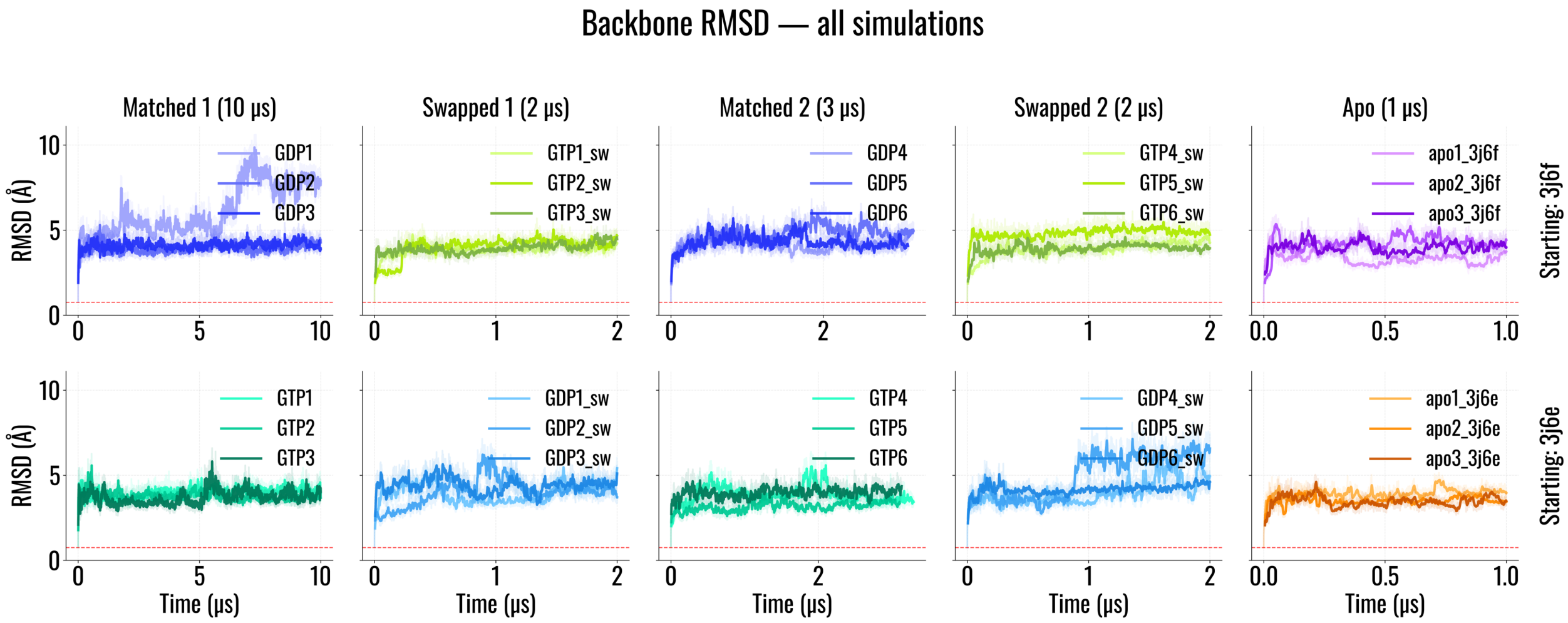

Figure S3: Backbone RMSD of the whole dimer relative to the starting structure, for all 30 replicas. Each panel shows the three replicas of one simulation set. The bold line is a running average over 30 ns and the transparent line of the same color is the raw trace. Columns correspond to the simulation sets (matched, swapped and apo, with their lengths given in the column headers) and rows to the starting structure (3J6F, top; 3J6E, bottom). The dashed red line marks the backbone RMSD between the two experimental starting structures (0.75Å). Note the different time axes across columns (10, 2, 3, 2 and 1 μs). Overall, in two simulations the nucleotide left the β-subunit binding pocket, most notably in GDP1, where the consequences of nucleotide dissociation are discussed in the main text. In GTP4, the nucleotide also dissociated after approximately 2.5 μs. However, no major conformational bias was observed. We also verified that frames corresponding to these dissociated states were not included in the subsequent network analysis.

SIV Calculation of the bending angles

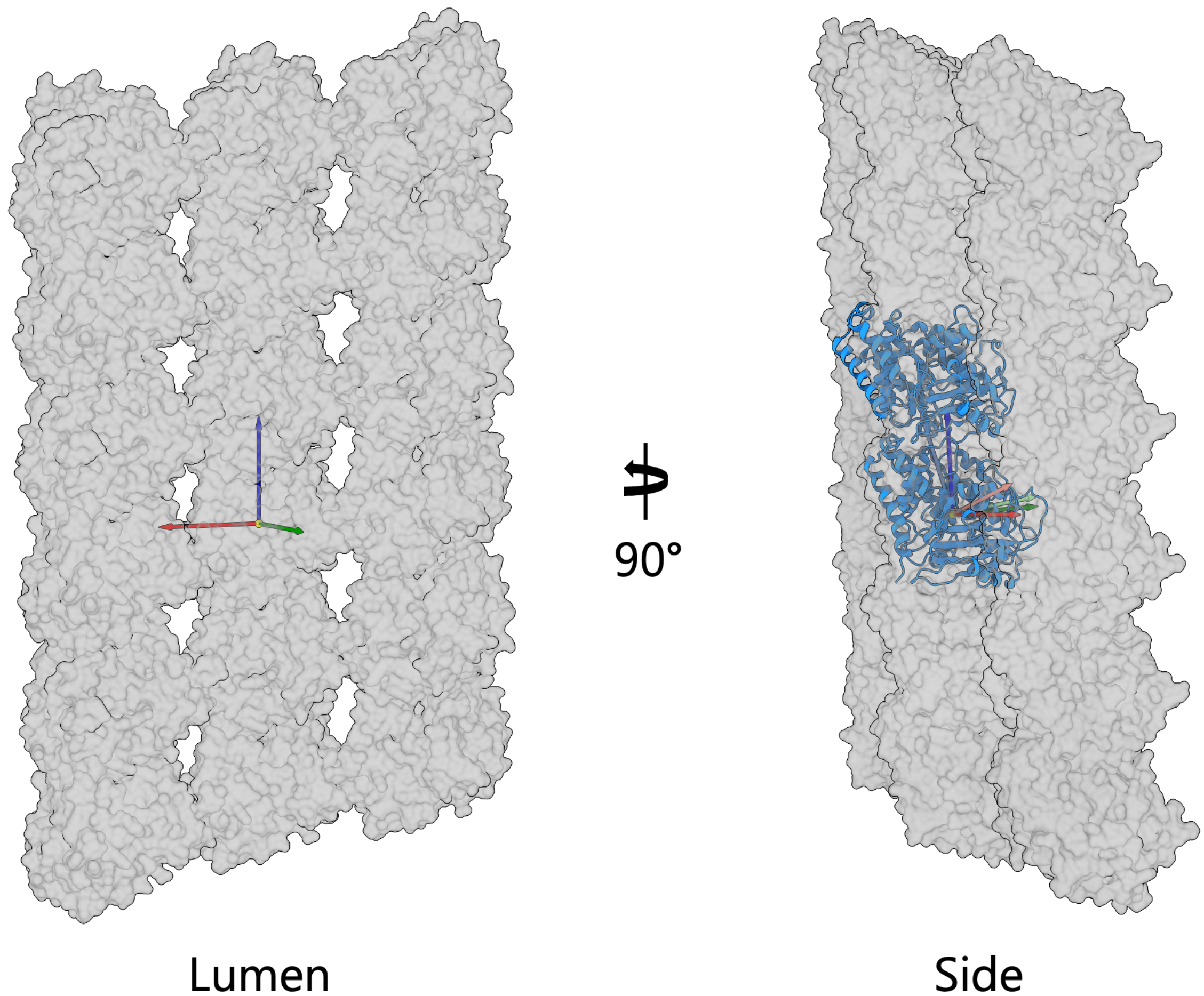

Figure S4: Definition of the reference coordinate system used for the calculation of intra-dimer bending angles. The coordinate axes are defined based on the geometry of the microtubule lattice (PDB ID 3J6F). **Left:** Microtubule fragment with xyz unit vectors as seen from the lumen (x - red, y - green, and z - blue). **Right:** Rotated microtubule fragment showing the tubulin dimer (blue cartoon) aligned to the β-subunit position within the lattice with XYZ unit vectors.

SV Quality of clustering

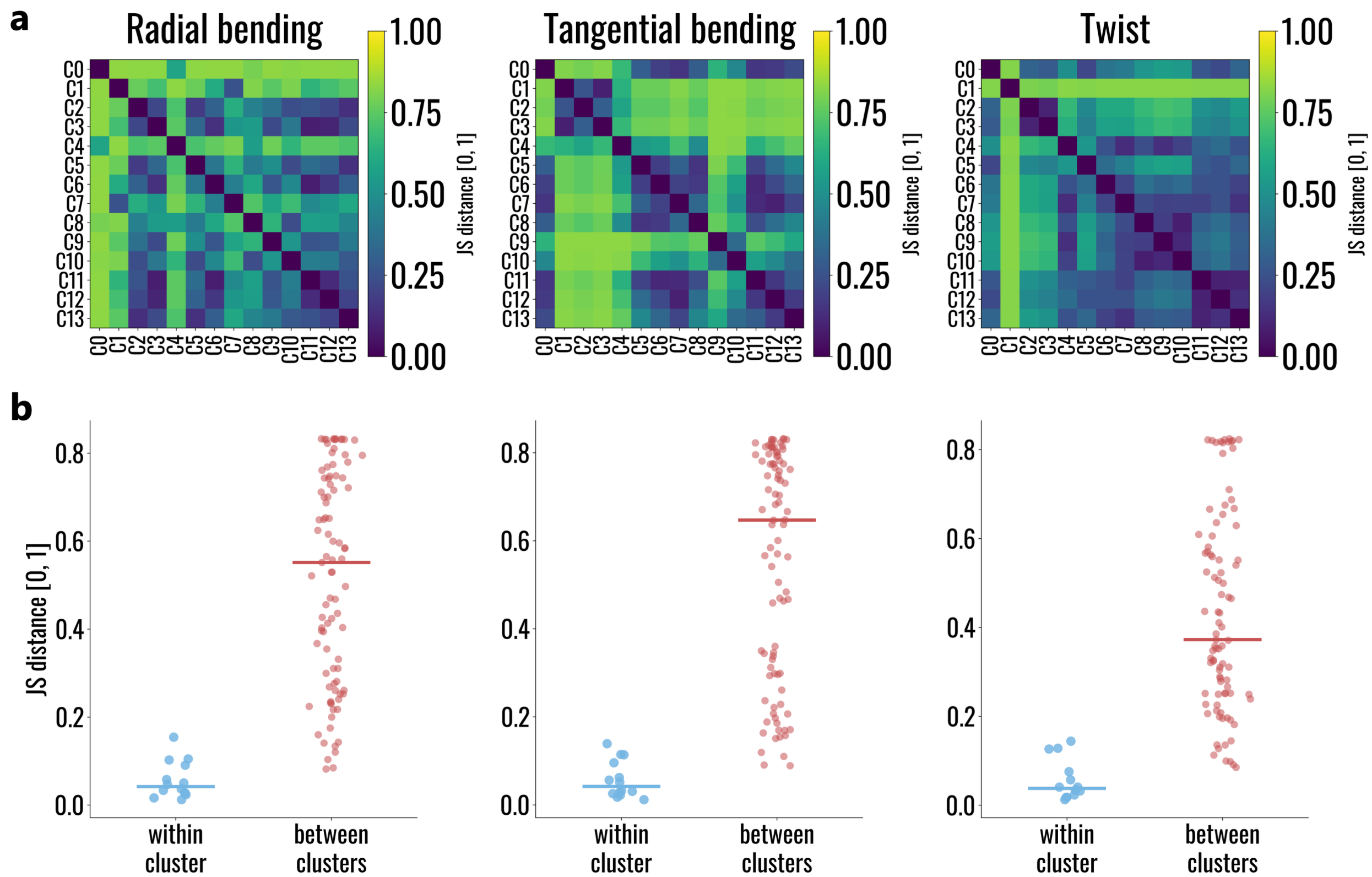

Figure S5: Validation of the clustering through intradimer bending angles. For each of the three intradimer bending angles (radial, tangential, and twist), the distributions in different clusters were compared using the Jensen–Shannon distance. The Jensen–Shannon distance quantifies the similarity between two probability distributions, with a value of 0 indicating identical distributions and larger values indicating greater dissimilarity. **a** Pairwise Jensen–Shannon distances between the angle distributions of every pair of clusters, shown as one heatmap per angle. **b** Distribution of Jensen–Shannon distances separated into within-cluster values (blue), obtained by comparing each cluster with a random split of itself into two halves, and between-cluster values (red), obtained by comparing every distinct pair of clusters. Horizontal bars indicate the median.

SVI Cluster composition

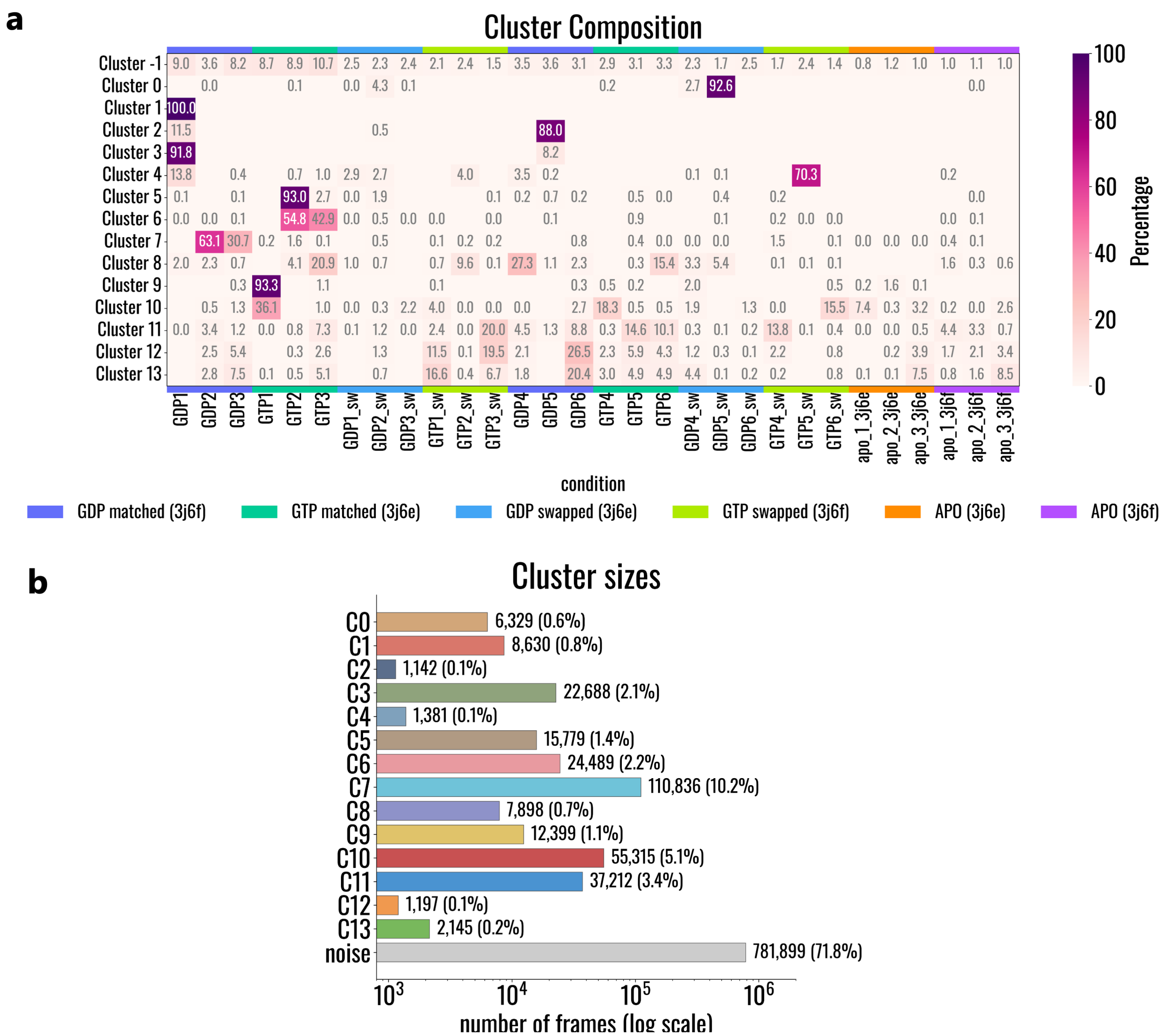

Figure S6: **a** Composition of HDBSCAN clusters by simulation replica, given as the percentage of each cluster's frames contributed by every replica (rows sum to 100%). The colored bars above and below the heatmap indicate the condition of each replica (GDP/GTP matched, GDP/GTP swapped, and APO). Cluster -1 represents the "noise" points that were not assigned to any specific cluster by the algorithm. **b** Number of frames assigned to each cluster on a logarithmic scale, with the fraction of the full dataset given in parentheses.

SVII Intradimer interface contacts across clusters

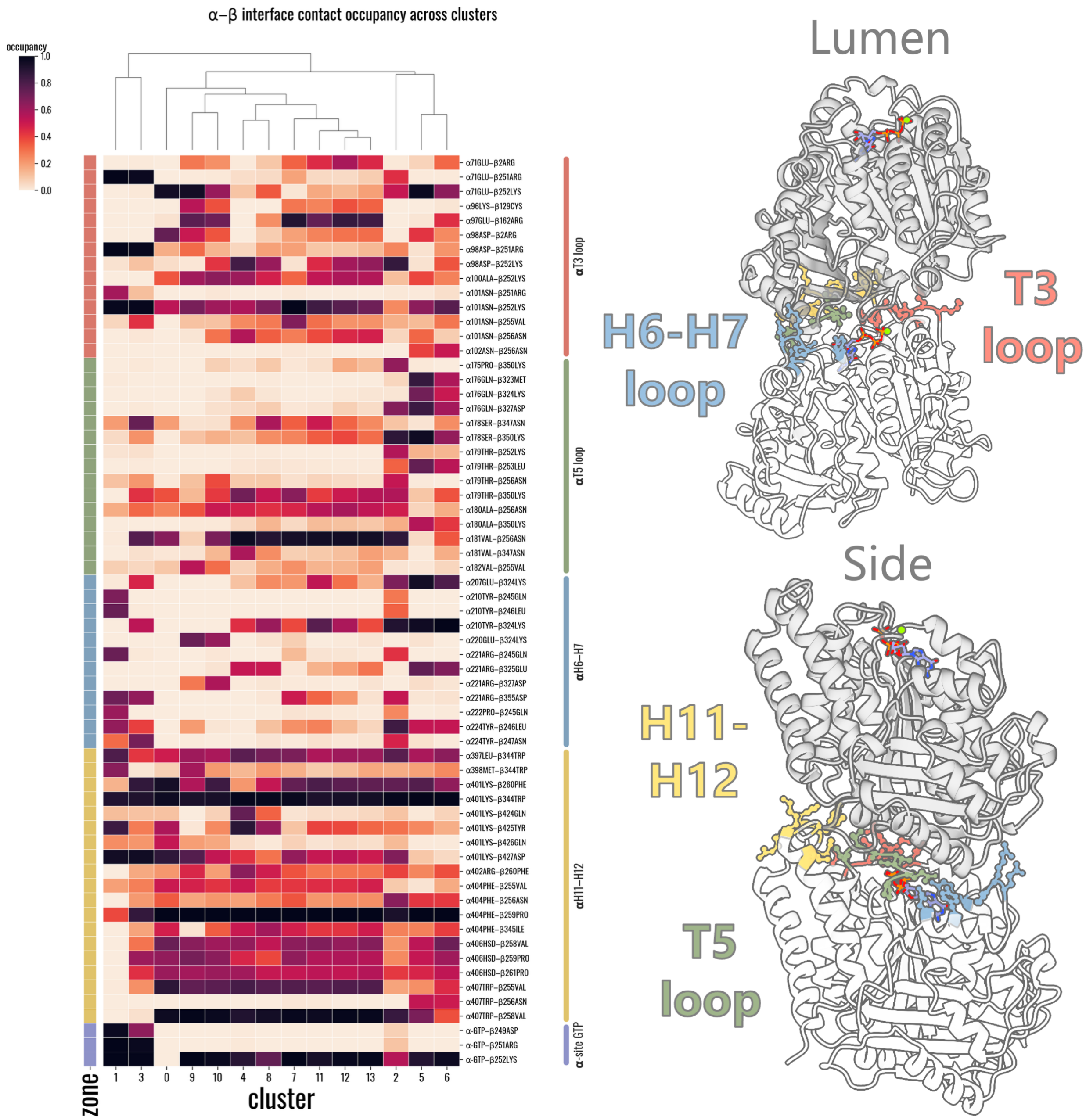

Figure S7: Occupancy of α–β interface contacts across the HDBSCAN clusters. Each row is an α–β residue contact and each column is one of the 14 clusters, the color encodes the contact occupancy, darker = higher. The contacts are grouped by the secondary-structure zone of α-tubulin they originate from (right and left color). On the right the contact zones are depicted on the initial structure of the tubulin dimer.

SVIII Cluster 8 vs Cluster 13 interface contacts

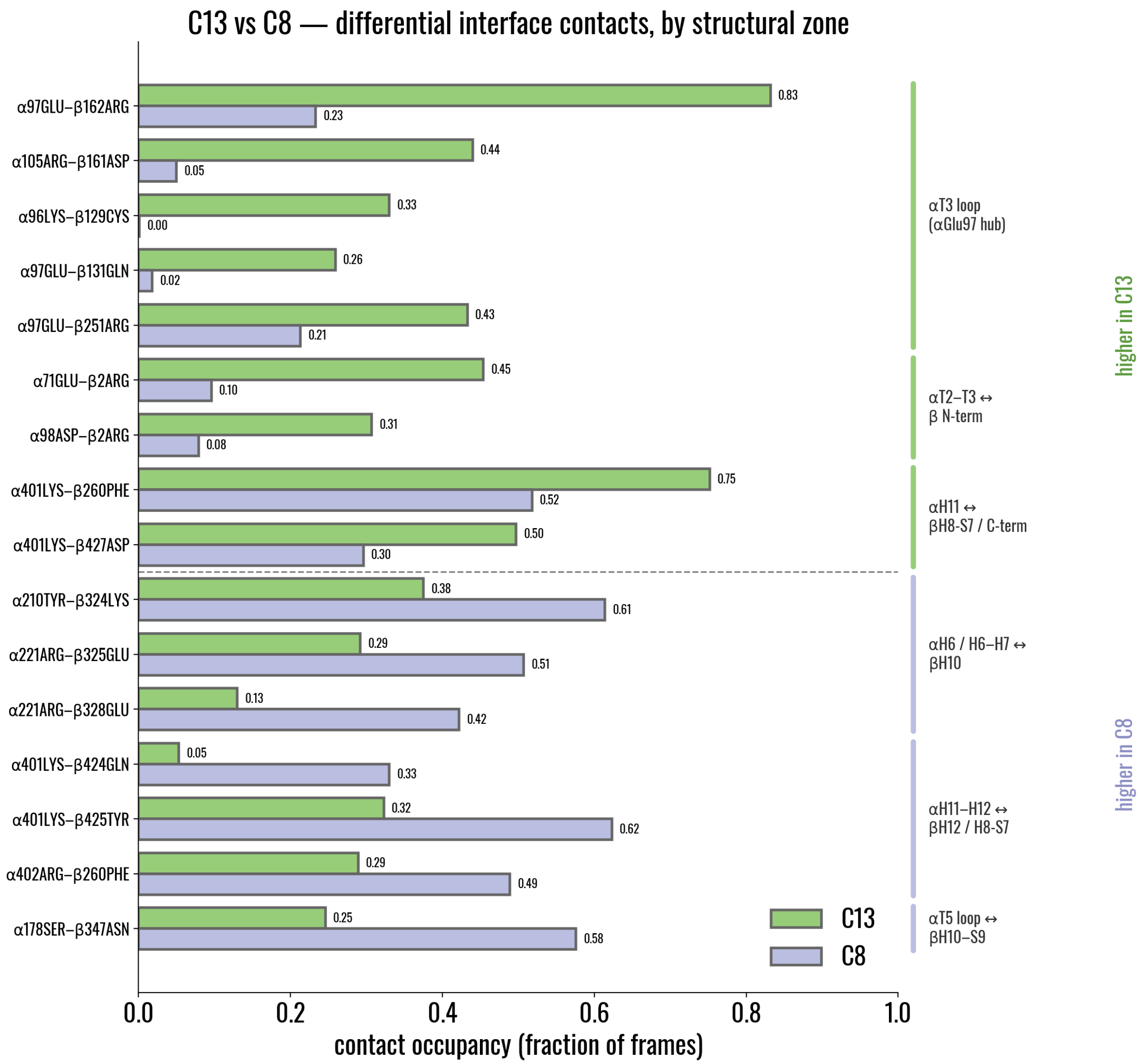

Figure S8: Difference in α–β interface contacts between Cluster 13 (green) and Cluster 8 (purple). Bars give the contact occupancy for a set of interface contacts that distinguish the two clusters. Contacts are grouped by the structural zone they belong to (right color strip) and split into those with higher occupancy in Cluster 13 (top) and in Cluster 8 (bottom).

SIX Exemplars

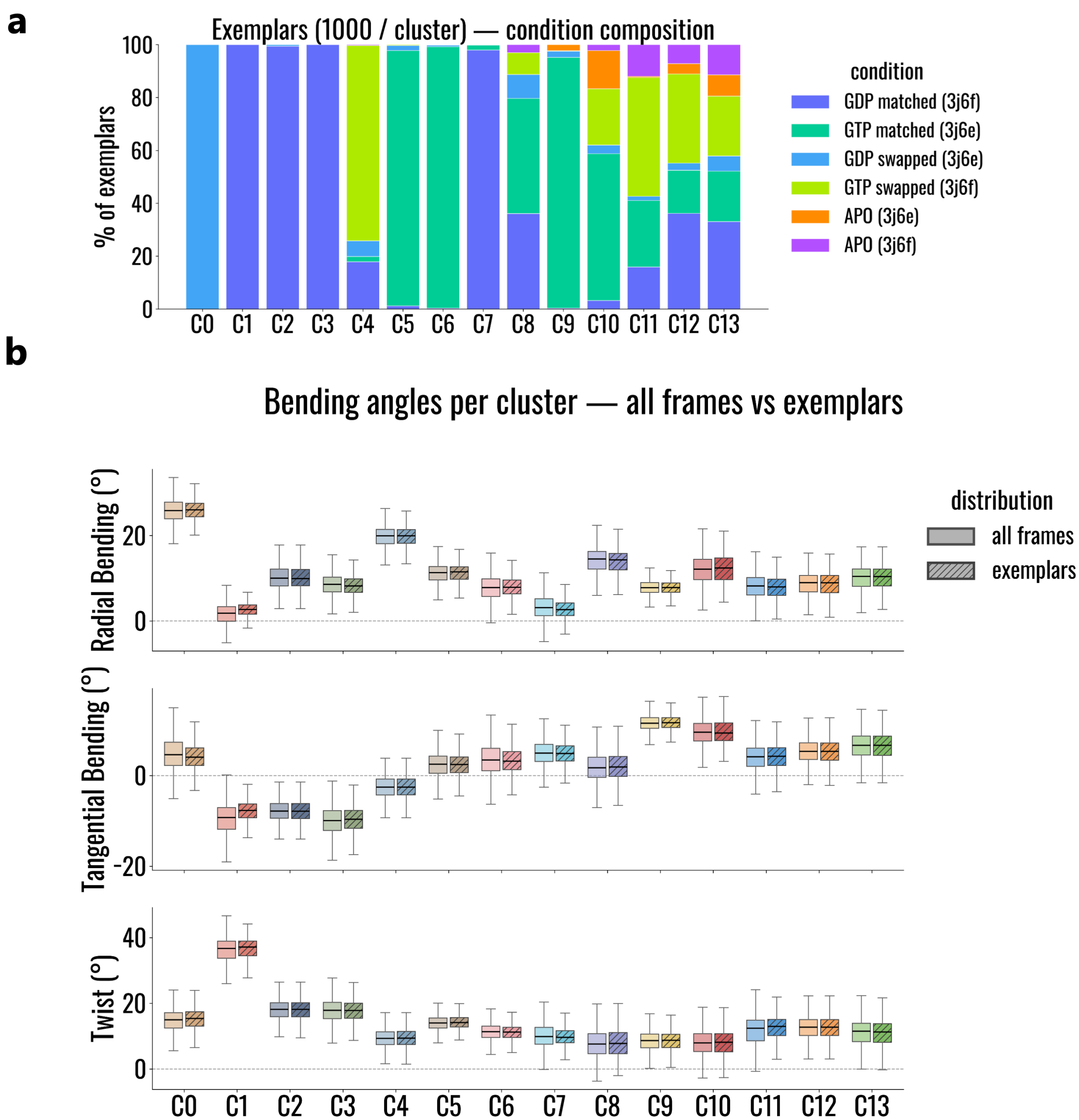

Figure S9: **a** Condition composition of each cluster's exemplars (1000 per cluster). **b** Per-cluster distributions of the radial, tangential and twist bending angles for all frames (solid) versus exemplars (hatched)

Table S1: Composition of the exemplar sets of clusters 8 and 13 (without apo).

		Exemplar frames	
Condition	Starting structure	Cluster 8	Cluster 13
GDP matched	3J6F	361	331
GTP matched	3J6E	436	191
GDP swapped	3J6E	90	57
GTP swapped	3J6F	83	226

SX Raw Betweenness centralities

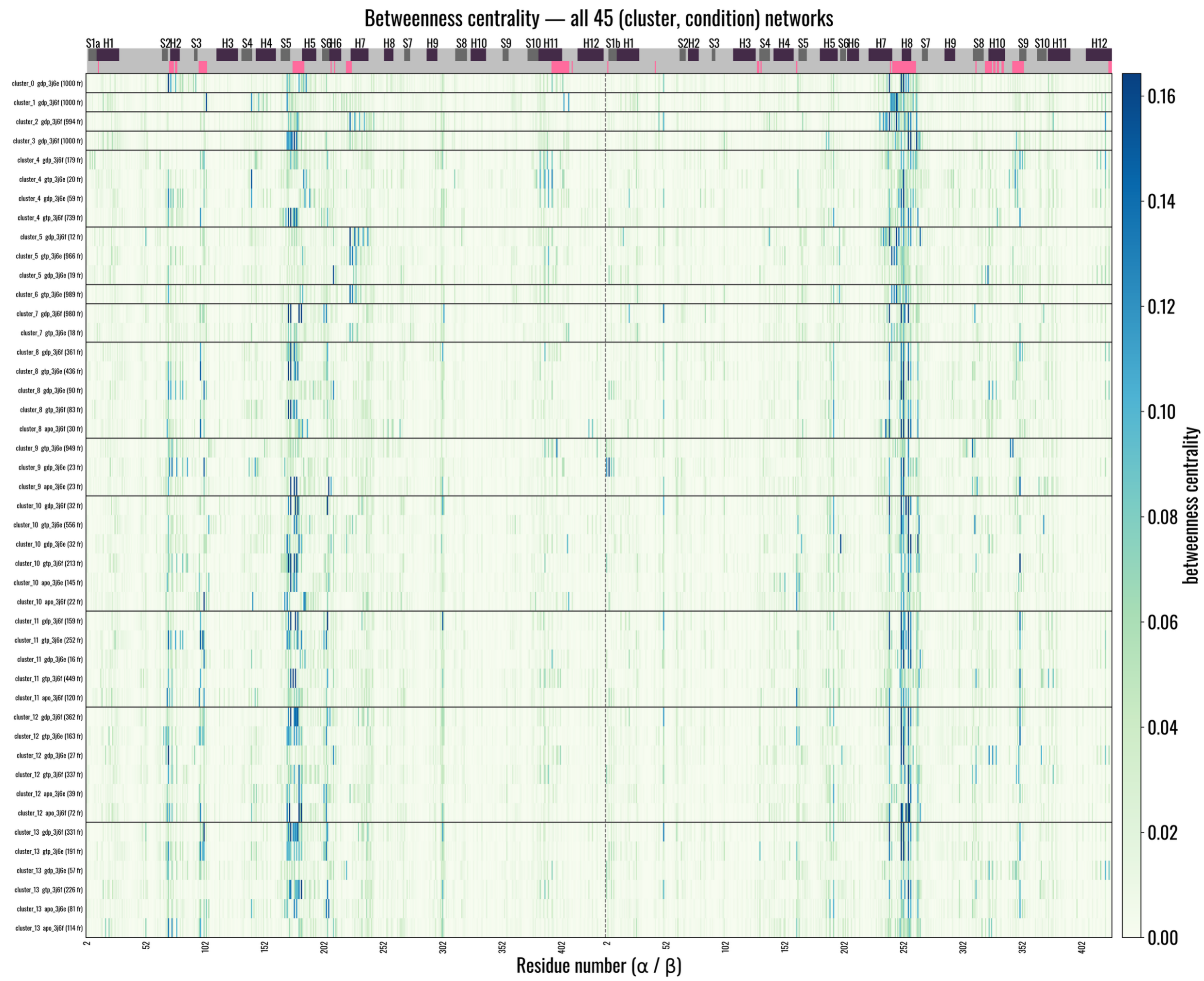

Figure S10: Residue betweenness centrality for all 45 (cluster, condition) networks. Each row is one network. The color gives the betweenness centrality of each residue where darker means higher. Rows are grouped by cluster (horizontal lines) and, within a cluster they are ordered by condition. The bars at the top show the secondary-structure elements of the two subunits and, in pink, the residues at the α–β interface.

SXI Source and Sink Residues

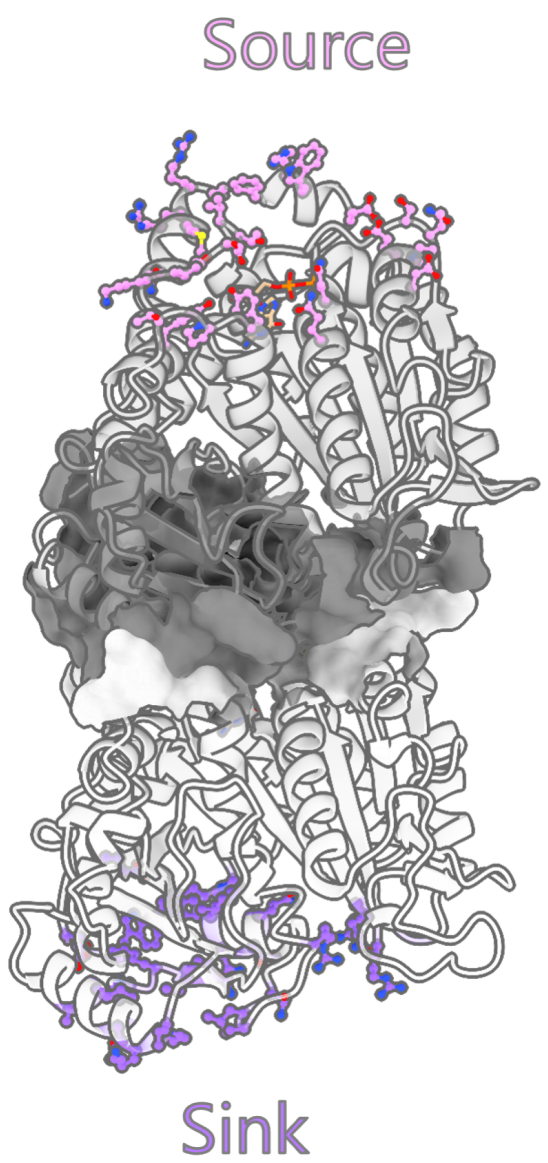

Figure S11: Structure of the tubulin dimer with the source and sink residues shown as atoms and the intradimer interface shown as a surface.

Table S2: Source and Sink Residues

Set	Residues
Source Residues	β11GLN, β15GLN, β69GLU, β70PRO, β71GLY, β74ASP, β94GLN, β95SER, β98GLY, β174LYS, β175VAL, β176SER, β177ASP, β178THR, β179VAL, β208TYR, β218THR, β220PRO, β222TYR, β384GLN, β388MET, β390ARG, β391ARG, β393ALA, β394PHE, β396HIS, β397TRP, β401GLU
Sink Residues	α2ARG, α131GLY, α133GLN, α163LYS, α243ARG, α247ALA, α248LEU, α249ASN, α256GLN, α257THR, α258ASN, α259LEU, α260VAL, α261PRO, α262TYR, α263PRO, α324VAL, α325PRO, α326LYS, α329ASN, α332ILE, α333ALA, α345ASP, α346TRP, α348PRO, α350GLY, α351PHE, α352LYS, α434GLU, α439SER

SXII Alternative dimensionality reduction techniques

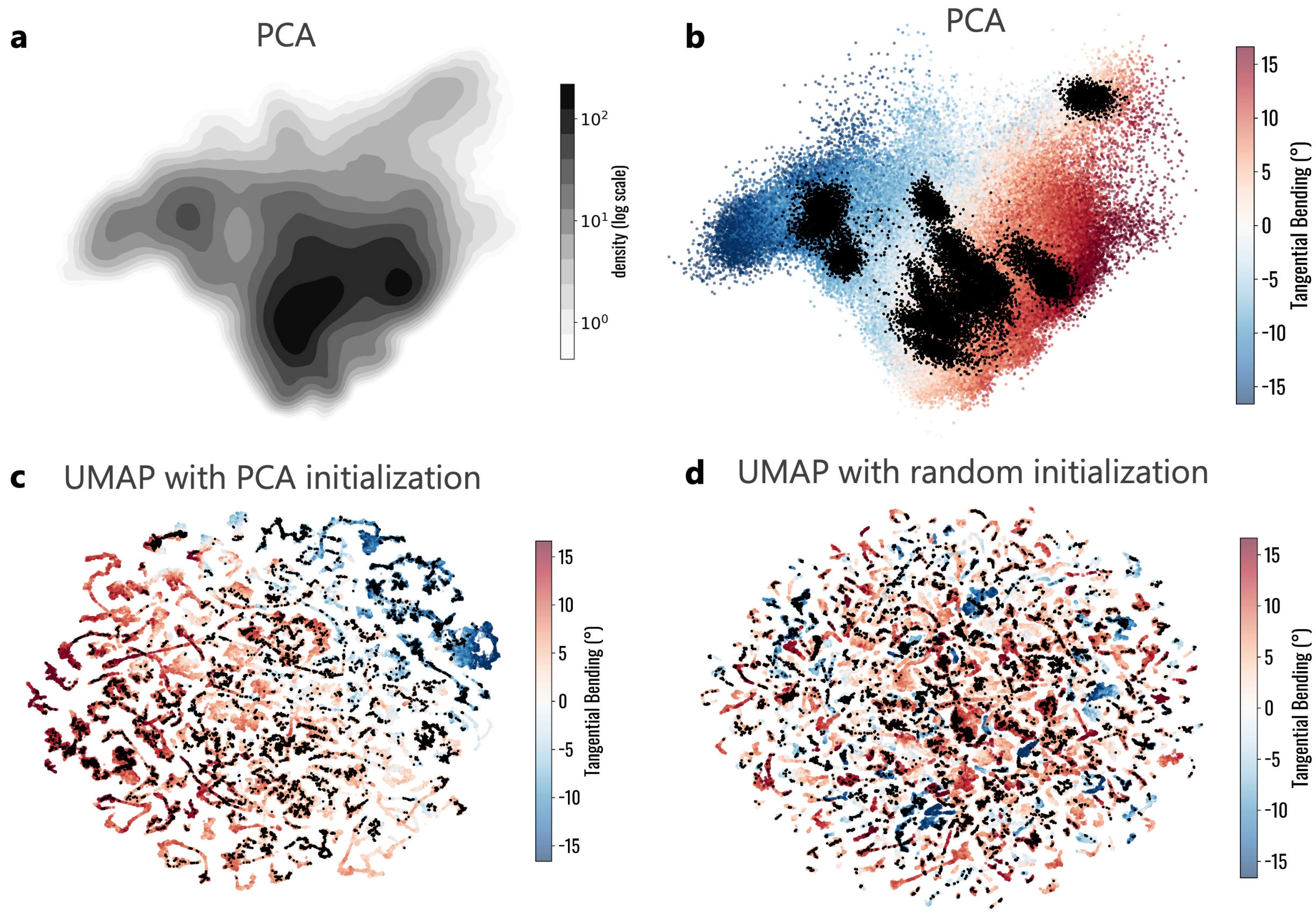

Figure S12: Comparison of alternative embeddings: **a** PCA projection colored by density. **b** PCA projection colored by the tangential bending of the dimer; black points are the cluster exemplars from EncoderMap. One observes that PCA (as a linear dimensionality reduction method) recovers the global bending behavior of the dimer and gives a smooth, interpretable projection similar to the EncoderMap two-dimensional landscape. However, as a linear projection it does not fully resolve the states found by EncoderMap. The EncoderMap cluster exemplars (black points in panel b), which represent distinct dimer geometries, are collapsed onto each other in the PCA projection.

c,d UMAP (`n_neighbors` = 200, `min_dist` = 0.5) colored by the tangential bending, with EncoderMap cluster exemplars shown as black points. The initialization is `pca` in **c** and `random` in **d**. UMAP, another non-linear dimensionality-reduction technique, places strong emphasis on the local structure in the data. It breaks the landscape into many small, disconnected islands, which does not aid interpretation of the landscape. Moreover, the layout strongly depends on the initialization method. With PCA initialization (panel **c**), the landscape inherits the smooth tangential bending gradient, but the fine-grained arrangement of the map is disrupted. With random initialization (panel **d**), any global structure is lost and the landscape is even harder to interpret.
